# Network pharmacology study on the mechanism of *Curcumae Rhizoma* in the treatment of non-small cell lung cancer

**DOI:** 10.1097/MD.0000000000042366

**Published:** 2025-05-09

**Authors:** Zhirui Yang, Mingquan Wu, Xin Zhou, Jin Luo, Yi Liu, Lin Li

**Affiliations:** a Department of Nuclear Medicine, Chengdu Second People’s Hospital, Chengdu, Sichuan, China; b Department of Pharmacy, Sichuan Orthopedic Hospital, Chengdu, Sichuan, China.

**Keywords:** bioactive compounds, *Curcumae Rhizoma*, network pharmacology, non-small cell lung cancer, underlying mechanism

## Abstract

Non-small cell lung cancer (NSCLC) poses a significant threat to public health worldwide. *Curcumae Rhizoma* (CR) has potent therapeutic potential in different cancers. However, the mechanism of CR treating NSCLC remains unclear. In this study, a network pharmacology-based strategy is followed to address the issue. The targets related to CR or NSCLC were obtained from multiple online public databases. Compound-target network was constructed using Cytoscape. Protein–protein interaction (PPI) was analyzed by STRING. Key transcription factors were explored in TRRUST. Gene ontology (GO) function and Kyoto encyclopedia of genes and genomes (KEGG) pathway enrichment analysis were accomplished in Metascape. The druglikeness of compounds was tested in Molinspiration Cheminformatics Software. Autodock Vina was used for molecular docking. Molecular dynamic (MD) simulation was performed using Gromacs. There were 104 overlapped targets considered as key targets of CR treating NSCLC. The key components of CR, including reynosin, (4S,5S)-13-hydroxygermacrone 4,5-epoxide, and (E)-1,7-bis(4-hydroxyphenyl)-6-hepten-3-one, were screened by topological parameters and bioactivity scores. Central clustered targets in PPI network (epidermal growth factor receptor [EGFR], SRC, JAK2, and mitogen-activated protein kinase 3 [MAPK3]) were identified as critical therapeutic targets of CR. GO and KEGG enrichment analysis suggested that therapeutic effect of CR on NSCLC involved various biological processes, cellular components, and molecular functions, and pathways in cancer, JAK-STAT signaling pathway, and p53 signaling pathway were strongly related. Molecular docking and MD simulation suggested that key compounds in CR had high binding affinity to critical NSCLC targets, like EGFR, JAK2, SRC, and MAPK3, with stable complexes formed. This study revealed key components and mechanism of CR treating NSCLC based on a network pharmacology-driven strategy, providing a reference for in-depth study on treating NSCLC.

## 1. Introduction

Lung cancer poses a significant threat to public health and is the most common cause of cancer-related death worldwide. As a leading cause of cancer-related morbidity, lung cancer contributes to 21% of the estimated deaths in the United States in 2022.^[1]^ Regrettably, there has been an upward trend in lung cancer incidence and mortality in China over the past few years, which has continued to rise. In 2020, an estimated 820,000 new diagnoses of lung cancer and 715,000 lung cancer-related deaths were reported in China, as revealed by global cancer statistics 2020.^[2,3]^ Among patients suffering from lung cancer, approximately 85% have a group of subtypes known as non-small cell lung cancer (NSCLC), which includes 2 major types: nonsquamous (including adenocarcinoma, large-cell carcinoma, and other cell types) and squamous cell (epidermoid) carcinoma.^[4,5]^ To improve the quality of life of patients, increasingly sophisticated therapies have been developed to improve the clinical outcomes. However, major challenges remain in the discovery of new promising drugs with clarified mechanisms, which would dramatically facilitate the development and application of targeted therapies against NSCLC, thus expanding the clinical benefit to a broader patient population.^[4]^

Chinese herbal medicines have provided an enormous collection of diverse compounds with potent bioactivities in the pharmaceutical industry, including the development of antineoplastic agents. Considered as a gift from nature, Chinese herbal medicines have been increasingly recognized owing to its adjuvant role in the treatment of cancer following chemotherapy and radiotherapy, *etc*., with the advantage of availability, efficacy, and minor side effects.^[6,7]^ *Curcumae Rhizoma* (CR), also known as *Ezhu* in Chinese, is the dry rhizome of *Curcuma phaeocaulis* Val., *Curcuma kwangsiensis* S. G. Lee et C. F. Liang, or *Curcuma wenyujin* Y. H. Chen et C. Ling. An increasing number of pharmacological studies have proven that CR exerts numerous bioactivities, including anticancer, anti-infection, anti-inflammation, hepatoprotection, and immunomodulation, among which anticancer bioactivity is the most reported, arousing great interest.^[8–11]^ The therapeutic potential of CR has been investigated in multiple cell lines involved in different types of cancers (e.g., gastric cancer, cervical cancer, breast cancer, lung cancer), of which main underlying mechanisms include stimulation of cell cycle arrest and cell apoptosis, inhibition of cell proliferation and metastasis, and recovery of suppressed proliferation of immune cells.^[12]^ Although the findings of these studies could be exciting, the current evidence of the mechanism underlying CR treatment in NSCLC is inadequate. Single compounds in CR are usually subjected to anticancer activity.^[13–15]^ Due to the chemical complexity and bioactivity diversity of Chinese herbal medicines, a strategy of depicting the comprehensive relationship between bioactive compounds and potential therapeutic targets should be considered.

The proposal of network pharmacology provides novel insights into the bioactive compounds and molecular mechanism of Chinese herbal medicines.^[16]^ With the rapid development of system biology and bioinformatics, network pharmacology is considered a valuable approach to a comprehensive understanding of drug actions. In the network pharmacology of herbs, various connections between herbal medicines and disease can be mathematically and computably represented into a network, highlighting a paradigm shift from the “one target, 1 drug” strategy to the “network target, multicomponent” strategy, which is helpful for elucidating the mechanism of Chinese herbal medicines.^[17]^ Therefore, in this study, we followed a network pharmacology-based strategy to identify the active compounds and explore the key targets associated with the treatment of NSCLC by CR, which would provide a reference for further in-depth study.

## 2. Materials and methods

Neither informed consent nor approval of the ethics committee is required for this study type, because no human or animal subjects or materials were use.

### 2.1. Chemical compounds in CR

Information on the chemical compounds of CR was obtained from Traditional Chinese Medicine Systems Pharmacology Database and Analysis Platform (TCMSP, https://old.tcmsp-e.com/tcmsp.php/) and literature retrieved from PubMed (https://pubmed.ncbi.nlm.nih.gov/) and China National Knowledge Infrastructure (https://www.cnki.net/).

### 2.2. Prediction of CR targets

Traditional Chinese Medicine Systems Pharmacology, Traditional Chinese Medicines Integrated Database (https://47.100.169.139/tcmid/), DrugBank (https://www.drugbank.com/), and SymMap (http://www.symmap.org/) were used to obtain the CR targets by uploading compound names. The simplified molecular input line entry system information of chemical compounds in CR was submitted to SwissTargetPrediction (http://www.swisstargetprediction.ch/) to predict the potential targets of CR.

### 2.3. Non-small cell lung cancer-related target genes

The target genes of NSCLC were retrieved from Online Mendelian Inheritance in Man (https://omim.org/), GeneCards (https://www.genecards.org/), and Comparative Toxicogenomics Database (https://ctdbase.org/). Standardization of the gene name and the species as “Homo sapiens” was performed using UniProt (https://www.uniprot.org/). The keyword was set as “non-small cell lung cancer.” The overlapping genes of CR and NSCLC-related targets which were identified by VENNY 2.1 online tool (https://bioinfogp.cnb.csic.es/tools/venny/) were considered as the key potential target genes for the treatment of NSCLC.

### 2.4. Construction of compound-target network

The compound-target network was constructed for the 55 compounds in CR and their potential targets in treating NSCLC using Cytoscape 3.9.1.^[18]^ The NetworkAnalyzer was used to calculate crucial topological parameters, including betweenness centrality (BC), closeness centrality (CC), and degree, to indicate the importance of compounds in the network.

### 2.5. Construction and analysis of protein–protein interaction network

The relationship between 104 potential targets was dissected using STRING 11.5 (https://cn.string-db.org/). “Homo sapiens” was set in the organisms. The network was built in Cytoscape 3.9.1 and the plugin CytoNCA was used to calculate the BC, CC, and Degree. The densely connected region in the network was discovered using the Molecular Complex Detection plugin.

### 2.6. Key transcription factors analysis of targets

The key transcription factors (TF) associated with the critical targets of CR treating NSCLC were explored in Transcriptional Regulatory Relationships Unraveled by Sentence-based Text mining (TRRUST, version 2, https://www.grnpedia.org/trrust/). Critical targets of CR were input with the species set as “Human.” The top 10 transcription factors ranked by the number of overlapped genes and *P*-value were selected to construct the transcription factor-target network using Cytoscape 3.9.1.

### 2.7. Gene ontology enrichment and Kyoto Encyclopedia of Genes and Genomes pathway enrichment analyses

Gene ontology function enrichment and KEGG pathway enrichment analyses were performed using Metascape (https://metascape.org/gp/index.html#/main/step1). The minimal overlap was set as 3, the *P*-value cutoff was .01, and the minimal enrichment factor was 1.5. The *P*-values were calculated based on the accumulative hypergeometric distribution, and *q* values were calculated using the Banjamini–Hochberg procedure to account for multiple testing. The most statistically significant term within a cluster was chosen to represent the cluster and displayed.

### 2.8. Molecular docking

The druglikeness of the top 20 compounds ranked by topological parameters in the compound-target network was tested using Molinspiration Cheminformatics Software (https://www.molinspiration.com/). The top 5 compounds were selected for docking based on their bioactivity scores. The structure of the chemical compounds of CR in sdf format was downloaded from PubChem (https://pubchem.ncbi.nlm.nih.gov/) or constructed in ChemDraw Professional 17.0 and converted into pdb format using Open Babel 3.1.1. The structure of the targets was downloaded from the RCSB Protein Data Bank (https://www.rcsb.org/) in pdb format. Docking was performed using AutoDock Vina. The docked complex with the highest affinity was further analyzed by BIOVIA Discovery Studio 4.5 Visualizer and visualized by PyMOL (version 2.5.2).

### 2.9. Molecular dynamic simulation

Molecular dynamic (MD) simulation was performed using Gromacs (2022.5) with Amber99sb-ildn force field to test the binding mode and energy of docked compound-target complex. A water box with a minimum distance of 1.2 nm between the protein atoms and the box edges was established. Ion auto-equilibrium system was added. Interaction of static electricity was analyzed using Particle-mesh Ewald method. Energy minimization was performed using a 50,000-step steepest descent method. The Coulomb force intercept and van der Waals radius intercept were both 1 nm, and the system was equilibrated in the canonical ensemble and isothermal isobaric system, and then MDs simulations were performed for 100 ns at room temperature and pressure. The cutoff distance for non-bonds was set to 10 Å. Room temperature was set using V-rescale method and pressure of 1.0 bar was set using Berendsen method during simulation process. The binding free energy was calculated via the Molecular Mechanics Poisson-Boltzmann Surface Area (MM/PBSA) method.

## 3. Results

### 3.1. Targets of CR in the treatment of NSCLC

**Figure 1.**
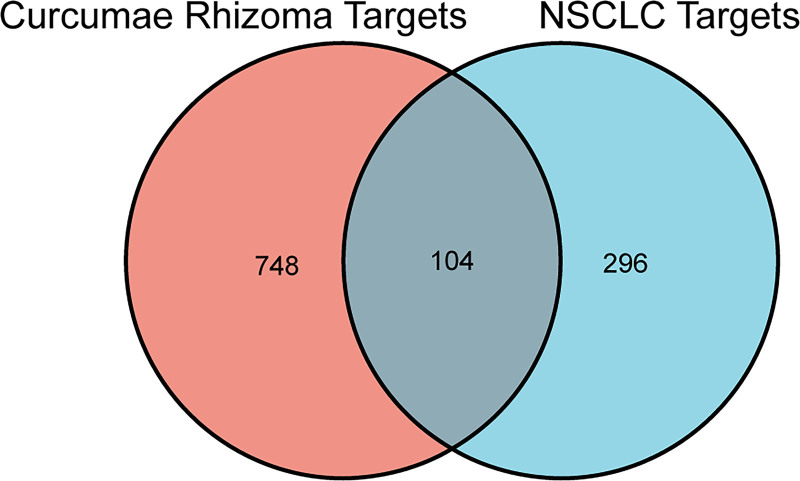
Venn diagram depicting common targets between *Curcumae Rhizoma* and (NSCLC). NSCLC = non-small cell lung cancer.

Online databases were searched, and literature was retrieved to obtain information on the chemical compounds of CR, and 55 compounds were included for target prediction, many of which were terpenoids and diarylheptanoids (Fig. S1, Supplemental Digital Content, https://links.lww.com/MD/O850, which shows the representative compounds in CR). The Venn diagram suggested that 104 common targets of both CR and NSCLC were considered as critical potential targets of CR in the treatment of NSCLC (Fig. 1 and Table S1, Supplemental Digital Content, https://links.lww.com/MD/O851, which shows the key targets of CR in the treatment of NSCLC). Therefore, these 104 target genes were used for compound-target network construction and further analysis.

### 3.2. Compound-target network

**Figure 2.**
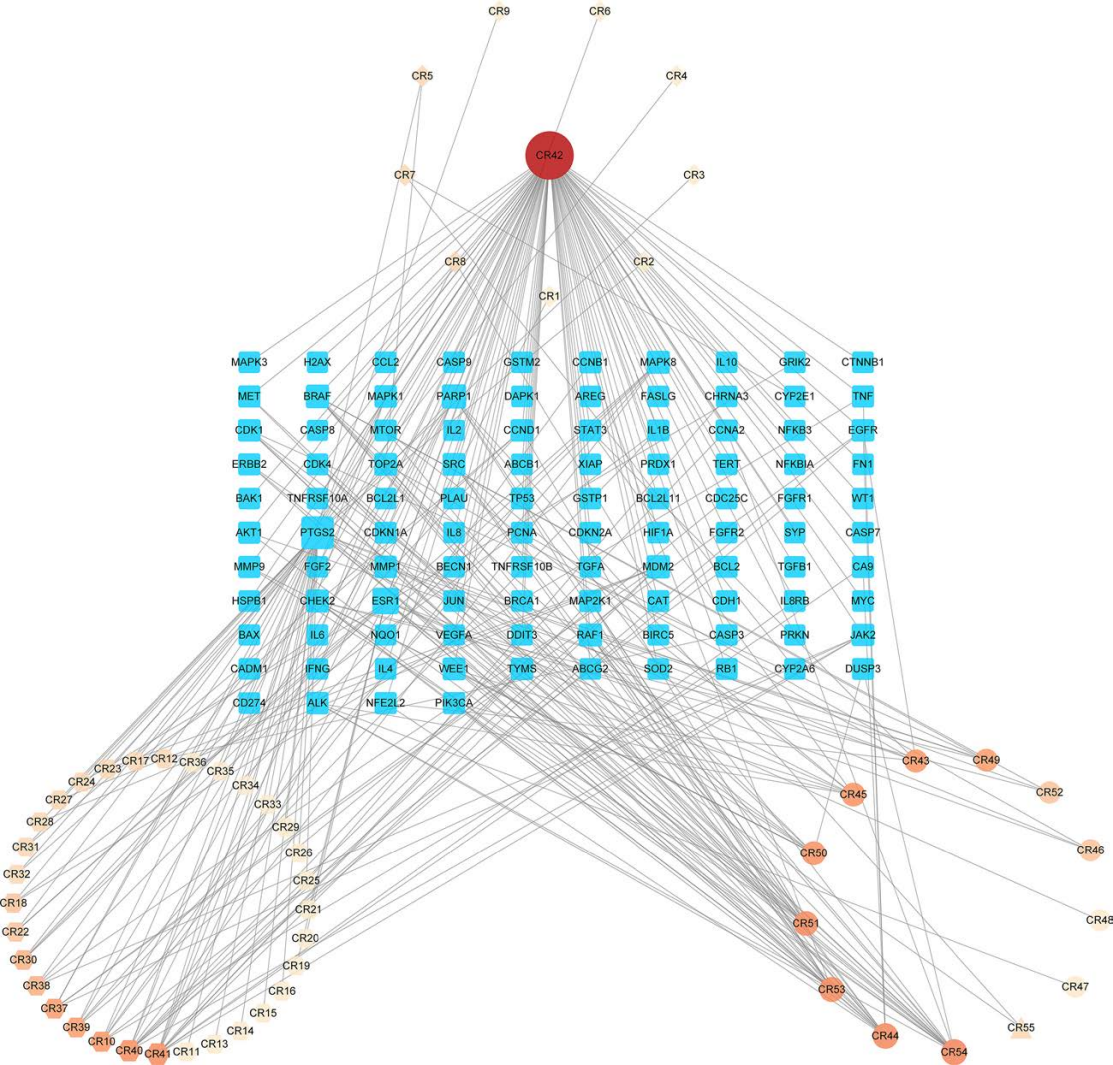
Compound-target network diagram. Diamonds represent monoterpenoids. Hexagons represent sesquiterpenes. Circles represent diarylheptanoids. Triangle represent 2-undecanone. Round rectangles represent common targets of *Curcumae Rhizoma* and non-small cell lung cancer. Size and color of nodes were proportional to the value of Degree.

A network depicting 55 CR compounds and 104 critical targets in treating NSCLC was developed by Cytoscape 3.9.1, which included 159 nodes (55 compound nodes and 104 target nodes) and 275 edges (Fig. 2). The NetworkAnalyzer was applied to calculate the crucial topological parameters, including BC, CC, and Degree (Table S2, Supplemental Digital Content, https://links.lww.com/MD/O852, which shows topological parameters of CR compounds in the compound-target network). The Degree value was proportional to the size and color of compound node to demonstrate the importance of node in the network. Notably, the network contained several compounds with higher values of Degree, BC, and CC, for example, curcumin, (4*S*,5*S*)-13-hydroxygermacrone 4,5-epoxide, (3*R*)-1-(3,4-dihydroxyphenyl)-7-(4-hydroxyphenyl)heptan-3-ol, (3*R*)-1-(3,4-dihydroxyphenyl)-7-phenyl-(6*E*)-6-hepten-3-ol, and (*E*)-1,7-bis(4-hydroxyphenyl)-6-hepten-3-one, suggesting their essential roles in the treatment of NSCLC.

### 3.3. Protein–protein interaction network

**Figure 3.**
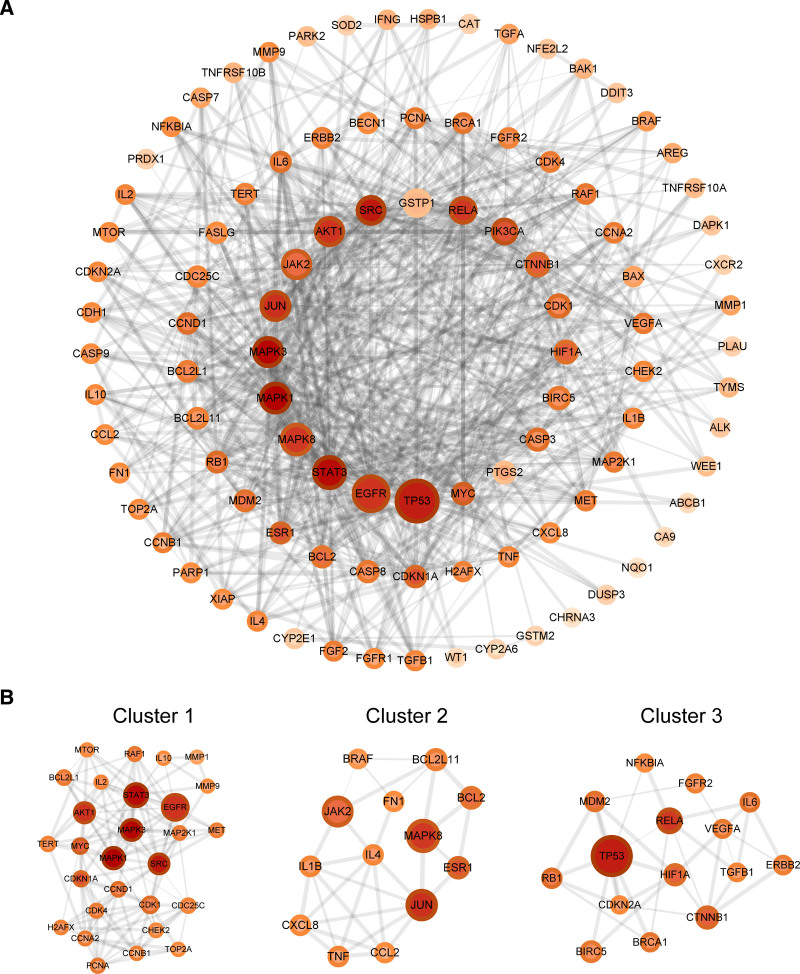
Protein-protein interaction network of key targets in the treatment of non-small cell lung cancer by *Curcumae Rhizoma*. (A) PPI network of 104 targets of *Curcumae Rhizoma* treating non-small cell lung cancer. (B) The top 3 central gene cluster identified by MCODE analysis. Node size was proportional to the value of BC. Node color was proportional to the value of CC. Border color of node was proportional to the value of degree. Edge width was proportional to the combined score calculated by STRING 11.5. BC = betweenness centrality, CC = closeness centrality, MCODE = Molecular Complex Detection, PPI = protein–protein interaction.

To explore the intrinsic relationship between the key targets of CR in the treatment of NSCLC, PPI analysis was performed using STRING. The PPI network developed using Cytoscape contained 99 nodes and 670 edges (Fig. 3A). CytoNCA was then applied to calculate the BC, CC, and Degree to discover the essential targets. The medians of the topological parameters were BC = 41.771, CC = 0.462, and Degree = 11. We found that some target genes had high topological parameters, such as cellular tumor antigen p53 (TP53), epidermal growth factor receptor (EGFR), tyrosine-protein kinase JAK2 (JAK2), signal transducer and activator of transcription 3 (STAT3), mitogen-activated protein kinase 8 (MAPK8), and proto-oncogene tyrosine-protein kinase Src (SRC), with their topological parameters being greater than the medians (Table 1). Molecular Complex Detection analysis, a useful approach to find highly interconnected regions in a network, showed 6 central gene clusters (Fig. 3B and Table 2). Intriguingly, we discovered that the top 10 key targets, including TP53, EGFR, JAK2, STAT3, MAPK8, mitogen-activated protein kinase 1, mitogen-activated protein kinase 3 (MAPK3), SRC, transcription factor Jun (JUN), and RAC-alpha serine/threonine-protein kinase (AKT1) were found in the top 3 central gene clusters, which consolidated their essential roles in the treatment of NSCLC by CR.

**Table 1:** The top 20 key genes in PPI network identified by BC, CC, and degree.

Genes	BC	CC	Degree
TP53	1105.2753	0.5868263	37
EGFR	829.0187	0.5697674	32
STAT3	658.4978	0.601227	38
MAPK8	599.9579	0.56647396	27
MAPK1	563.85754	0.6086956	39
MAPK3	527.67395	0.60493827	38
JUN	517.7306	0.57309943	32
JAK2	493.68707	0.5268817	26
AKT1	487.06064	0.5697674	31
SRC	469.93237	0.5903614	35
GSTP1	414.96002	0.4117647	5
RELA	375.34296	0.56647396	30
PIK3CA	330.85077	0.54444444	30
CTNNB1	250.70735	0.5355191	22
CDK1	221.86093	0.5051546	21
HIF1A	210.7281	0.5355191	20
BIRC5	182.94667	0.49	17
CASP3	176.9861	0.48756218	20
PTGS2	158.59677	0.44144145	5
MYC	153.94844	0.5355191	25

BC = betweenness centrality, CC = closeness centrality, PPI = protein–protein interaction.

**Table 2:** Central gene clusters of PPI network identified by MCODE analysis.

Cluster	Score	Nodes	Edges	Node IDs
1	9.333	28	126	CCND1, CHEK2, TOP2A, IL2, IL10, TERT, MMP9, PCNA, MAPK1, BCL2L1, AKT1, CDKN1A, MET, EGFR, CDC25C, SRC, CDK4, STAT3, H2AFX, CCNA2, MAPK3, MAP2K1, MTOR, CDK1, RAF1, MMP1, CCNB1, MYC
2	5	13	30	CCL2, JUN, FN1, JAK2, IL4, IL1B, ESR1, BRAF, BCL2L11, MAPK8, TNF, CXCL8, BCL2
3	4.714	15	33	IL6, RELA, RB1, NFKBIA, HIF1A, TP53, FGFR2, BRCA1, CDKN2A, ERBB2, TGFB1, VEGFA, MDM2, BIRC5, CTNNB1
4	4	4	6	GSTP1, CYP2E1, GSTM2, CYP2A6
5	3	3	3	CASP3, CASP7, PARP1
6	3	3	3	CASP8, TNFRSF10A, TNFRSF10B

MCODE = Molecular Complex Detection, PPI = protein–protein interaction.

### 3.4. Key transcription factor analysis

**Figure 4.**
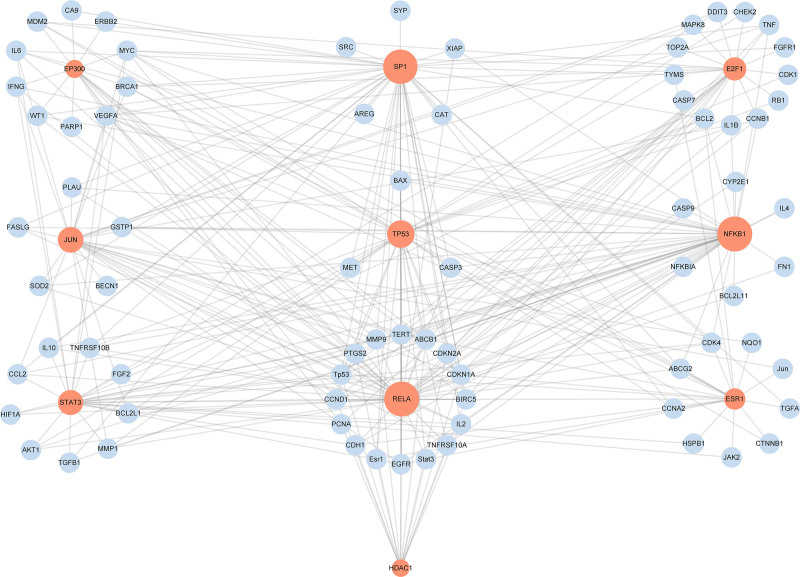
Network of the top 10 key TF and their related targets. Orange nodes represent TF and the node size was proportional to the number of overlapped genes. Sky blue nodes represent corresponding targets. TF = transcription factors.

In the PPI network, we found some transcription factors such as TP53, STAT3, JUN, and transcription factor p65 (RELA) which were involved in the treatment of NSCLC by CR. Thus, the potential role of transcription factors in the mechanisms underlying CR in NSCLC treatment was further investigated. By inputting 104 critical targets to TRRUST, a manually curated database of human and mouse transcriptional regulatory networks, the information of relevant transcription factors was acquired. Based on the number of overlapping genes, the top 10 transcription factors were selected to build the network, together with their corresponding targets (Fig. 4 and Table 3). Among them, TP53, JUN, STAT3, and RELA were also highlighted in the PPI analysis, suggesting their essential roles in the treatment of NSCLC via CR.

**Table 3:** The top 10 key TF correlated with targets of *Curcumae Rhizoma* in the treatment of non-small cell lung cancer.

TF	Overlapped gene number	*Q* value	*P*-value	Description
RELA	44	8.95 × 10^−51^	6.20 × 10^−53^	v-rel reticuloendotheliosis viral oncogene homolog A (avian)
NFKB1	44	8.95 × 10^−51^	8.45 × 10^−53^	nuclear factor of kappa light polypeptide gene enhancer in B-cells 1
SP1	43	1.20 × 10^−40^	1.70 × 10^−42^	Sp1 transcription factor
TP53	32	1.95 × 10^−40^	3.68 × 10^−42^	tumor protein p53
JUN	29	1.83 × 10^−36^	4.32 × 10^−38^	jun proto-oncogene
STAT3	28	2.21 × 10^−35^	6.24 × 10^−37^	signal transducer and activator of transcription 3 (acute-phase response factor)
ESR1	22	1.21 × 10^−31^	4.01×10^−33^	estrogen receptor 1
E2F1	25	7.13 × 10^−31^	2.69 × 10^−32^	E2F transcription factor 1
EP300	17	9.34 × 10^−25^	3.97 × 10^−26^	E1A binding protein p300
HDAC1	16	5.77 × 10^−21^	2.72 × 10^−22^	Histone deacetylase 1

TF = transcription factors.

### 3.5. Gene ontology enrichment and KEGG pathway enrichment analyses

**Figure 5.**
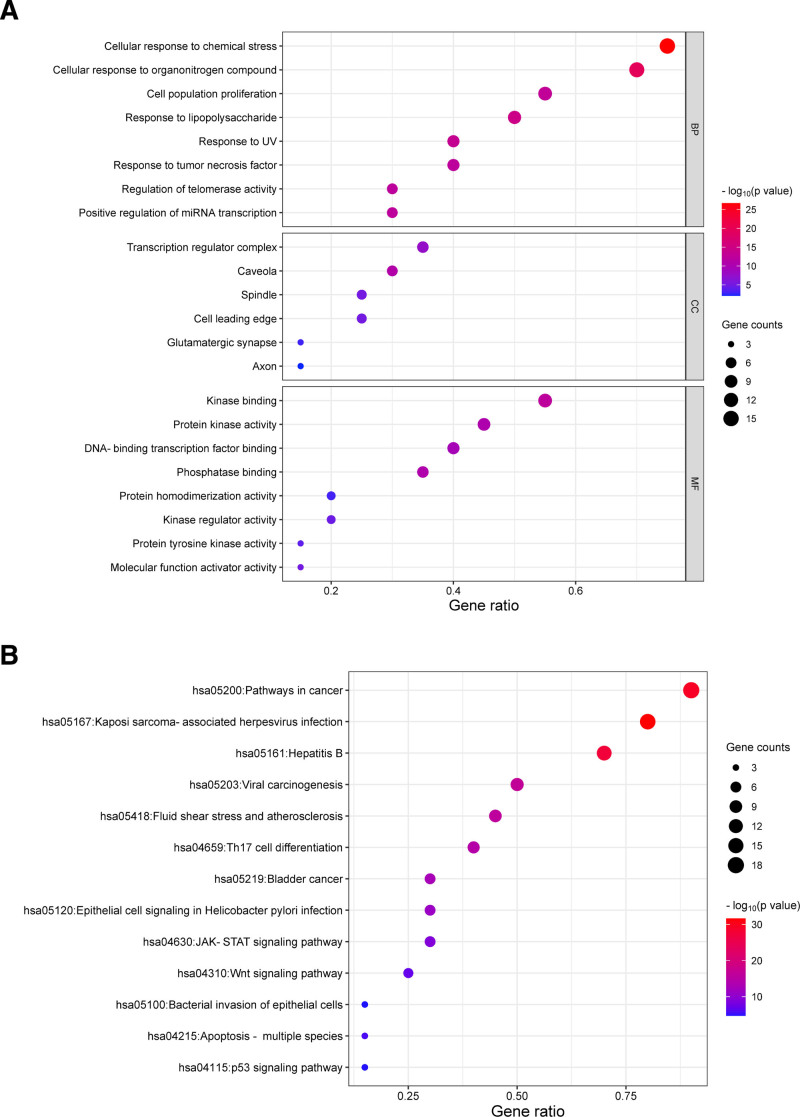
(A) Gene ontology enrichment analysis and (B) Kyoto encyclopedia of genes and genomes pathway enrichment analysis. BP = biological processes, CC = cellular components, MF = molecular functions.

The top 20 key target genes in the PPI analysis were selected for GO enrichment and KEGG pathway enrichment analyses. Metascape was used to clarify the functional annotation of the 20 key target genes. In the GO enrichment analysis, there were 416 entries in biological processes, 28 entries in cellular components, and 39 entries in molecular functions. The main biological processes involved in the CR treatment of NSCLC were cellular response to chemical stress, cellular response to organonitrogen compound, cell population proliferation, response to lipopolysaccharide, response to UV, response to tumor necrosis factor, regulation of telomerase activity, and positive regulation of miRNA transcription. The main cellular components were transcription regulator complex, caveola, spindle, cell leading edge, glutamatergic synapse, and axon. The main molecular functions included kinase binding, protein kinase activity, DNA-binding transcription factor binding, phosphatase binding, protein homodimerization activity, kinase regulator activity, protein tyrosine kinase activity, and molecular function activator activity (Fig. 5A). A total of 141 terms were acquired in KEGG pathway enrichment analysis. Key target genes were mainly enriched in the pathways in cancer, viral carcinogenesis, JAK-STAT signaling pathway, Wnt signaling pathway, p53 signaling pathway, and so forth (Fig. 5B). These signaling pathways might collaboratively function in the therapeutic effect of CR on NSCLC.

### 3.6. Molecular docking

**Figure 6.**
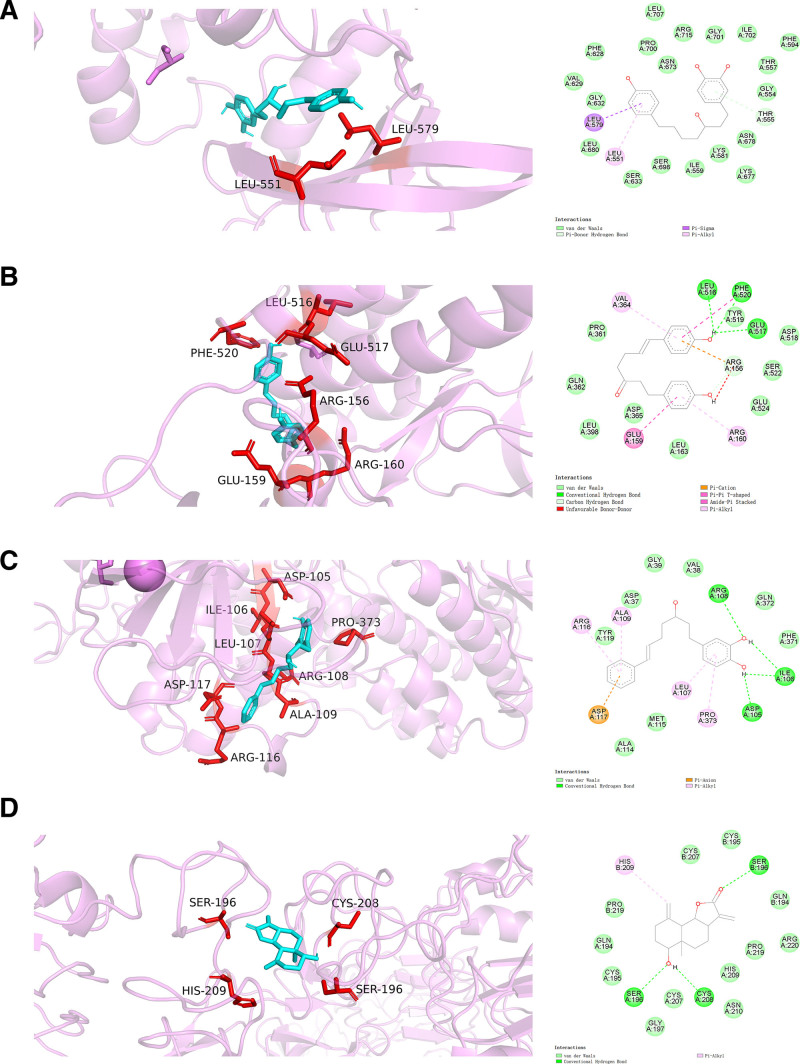
Representative diagrams of molecular docking between compounds in *Curcumae Rhizoma* and the targets. (A) JAK2 and (3*R*)-1-(3,4-dihydroxyphenyl)-7-(4-hydroxyphenyl)heptan-3-ol. (B) SRC and (*E*)-1,7-bis(4-hydroxyphenyl)-6-hepten-3-one. (C) MAPK3 and (3*R*)-1-(3,4-dihydroxyphenyl)-7-phenyl-(6*E*)-6-hepten-3-ol. (D) EGFR and reynosin. EGFR = epidermal growth factor receptor, JAK2 = tyrosine-protein kinase JAK2, MAPK3 = mitogen-activated protein kinase 3, SRC = proto-oncogene tyrosine-protein kinase Src.

**Figure 7.**
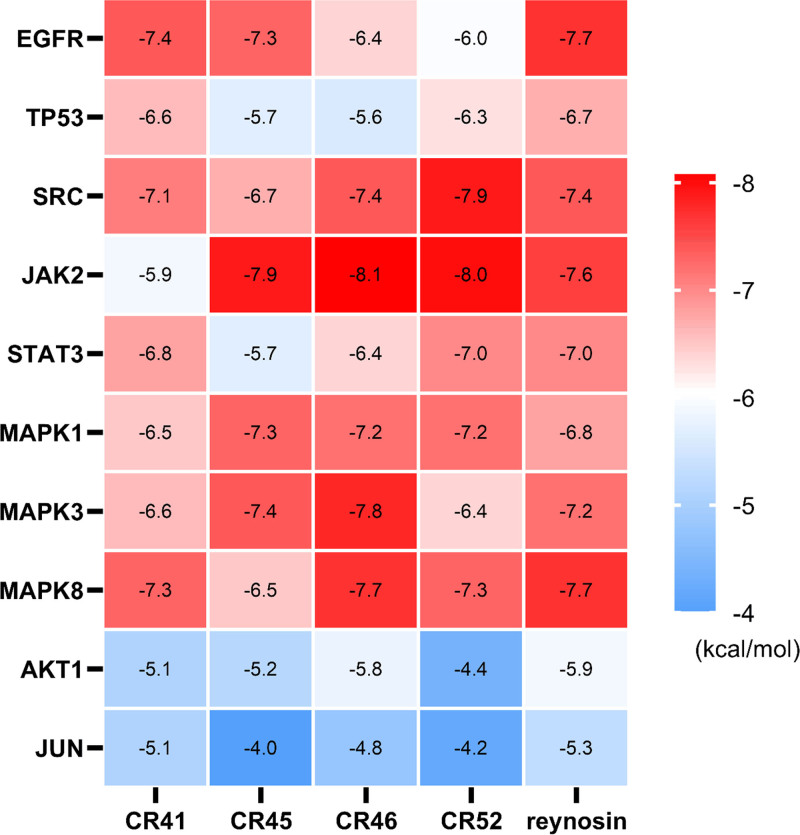
Heatmap of binding affinity. CR41, (4*S*,5*S*)-13-hydroxygermacrone 4,5-epoxide. CR45, (3*R*)-1-(3,4-dihydroxyphenyl)-7-(4-hydroxyphenyl)heptan-3-ol. CR46, (3*R*)-1-(3,4-dihydroxyphenyl)-7-phenyl-(6*E*)-6-hepten-3-ol. CR52, (*E*)-1,7-bis(4-hydroxyphenyl)-6-hepten-3-one.

In the compound-target network, the compounds in CR that played essential roles in treating NSCLC were identified by ranked topological parameters. To further discover the key compounds treating NSCLC, we input the top 20 compounds into Molinspiration Cheminformatics Software to test their druglikeness by calculating bioactivity scores. We found that 5 compounds, namely, reynosin (CR37), (4*S*,5*S*)-13-hydroxygermacrone 4,5-epoxide (CR41), (3*R*)-1-(3,4-dihydroxyphenyl)-7-(4-hydroxyphenyl)heptan-3-ol (CR45), (3*R*)-1-(3,4-dihydroxyphenyl)-7-phenyl-(6*E*)-6-hepten-3-ol (CR46), and (*E*)-1,7-bis(4-hydroxyphenyl)-6-hepten-3-one (CR52) had high scores, suggesting their potential value in drug development (Table 4). Thus, these 5 compounds were selected to analyze the binding affinity and interactions between the compounds and targets by docking with the top 10 key targets in the PPI network. We found high binding affinities and various interactions in some docked complexes (Figs. 6 and 7 and Figs. S2–S11, Supplemental Digital Content, https://links.lww.com/MD/O853, which shows the molecular docking of key targets and representative compounds in CR). For example, (3*R*)-1-(3,4-dihydroxyphenyl)-7-(4-hydroxyphenyl)heptan-3-ol bound strongly to JAK2 by forming pi-sigma hydrophobic bonds at Leu^551^ and Leu^579^. The hydroxyl groups of (*E*)-1,7-bis(4-hydroxyphenyl)-6-hepten-3-one generated conventional hydrogen bonds with Leu^516^, Glu^517^, and Phe^520^ in SRC. A pi-cation hydrogen bond and pi-pi T-shaped, amide-pi stacked, and pi-alkyl hydrophobic bonds were observed at Arg^156^, Glu^159^, Phe^520^, Arg^160^, and Val^364^. The complexes of MAPK3 and (3*R*)-1-(3,4-dihydroxyphenyl)-7-phenyl-(6*E*)-6-hepten-3-ol were stabilized by conventional hydrogen bonds generated between the 2 hydroxyl groups of the compound and Asp^105^, Ile^106^, and Arg^108^ of MAPK3. Pi-anion electrostatic force at Asp^117^ and pi-alkyl hydrophobic bonds at Ala^109^, Arg^116^, Leu^107^, and Pro^373^ were also observed. Conventional hydrogen bonds were formed between the hydroxyl and carbonyl group of reynosin and Ser^196^ and Cys^208^ of EGFR. It also formed a pi-alkyl hydrophobic bond at His^209^. These results proved that the reynosin and diarylheptanoids with a chemical structure similar to that of curcumin in CR had a high affinity to the critical targets of NSCLC, highlighting their potential therapeutic value in the treatment of NSCLC.

**Table 4:** Bioactivity scores of the top 5 critical compounds in *Curcumae Rhizoma*.

Compounds	GPCR ligand	Ion channel modulator	Kinase inhibitor	Nuclear receptor ligand	Protease inhibitor	Enzyme inhibitor
Reynosin	0.27	0.3	−0.33	1.18	−0.01	0.91
(3*R*)-1-(3,4-dihydroxyphenyl)-7-phenyl-(6*E*)-6-hepten-3-ol	0.37	0.32	0.16	0.54	0.24	0.48
(4*S*,5*S*)-13-hydroxygermacrone 4,5-epoxide	0.01	−0.2	−0.86	0.54	0.07	0.75
(3*R*)-1-(3,4-dihydroxyphenyl)-7-(4-hydroxyphenyl)heptan-3-ol	0.31	0.26	0.07	0.44	0.25	0.36
(*E*)-1,7-bis(4-hydroxyphenyl)-6-hepten-3-one	0.21	0.16	−0.17	0.39	0.12	0.35

GPCR = G-protein coupled receptors.

### 3.7. MD simulation

**Figure 8.**
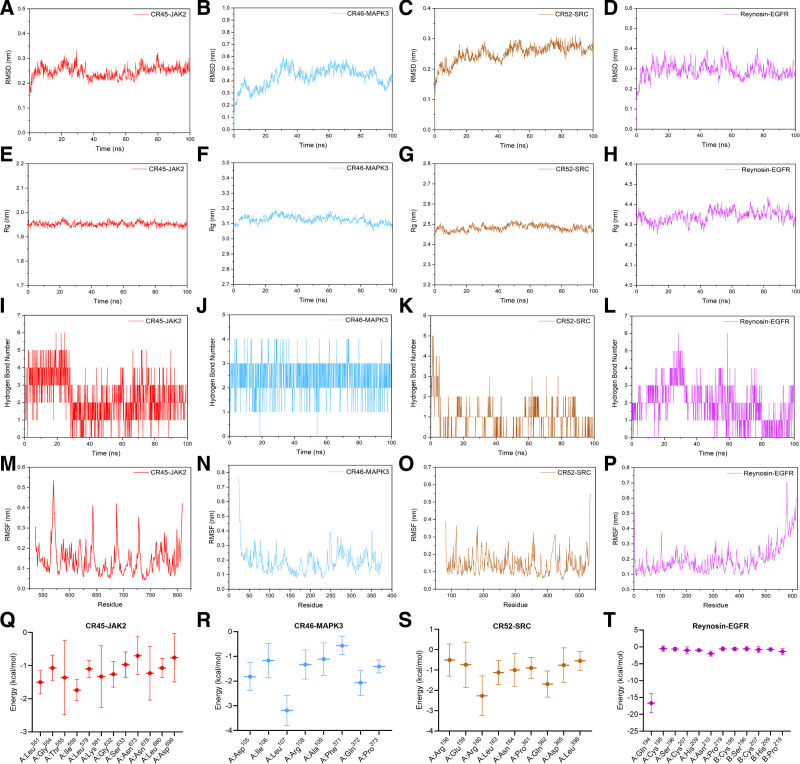
Molecular dynamic simulation. (A–D) The RMSD. (E–H) Rg. (I–L) The number of hydrogen bonds. (M–P) The RMSF. (Q–T) The energy decomposition diagram of amino acids. Rg = radius of gyration, RMSD = root mean square deviation, RMSF = root mean square function.

Molecular dynamic simulation was further employed to assess the binding characteristics between compounds and their targets and examine the stability of the docked complexes. The root mean square deviation (RMSD) can assess the conformational stability of the compound-target complex and indicate whether the simulation is in equilibrium. It was shown that the RMSDs of CR45-JAK2, CR46-MAPK3, CR52-SRC, and reynosin-EGFR complexes were all <0.6 nm, while the complexes reached dynamic equilibrium rapidly, which suggested that the binding between compounds and target protein was relatively stable. No break in the RMSD curve was observed, suggesting that the compounds and target proteins could be firmly attached without any dissociation during the simulation (Fig. 8A–D).

The radius of gyration (Rg) is important for quantifying the protein structural variability during MD simulation. Low and stable Rg values of 4 compound-target complexes indicated that these systems were compact and rigid, among which the structure of CR45-JAK2 complex was the most rigid with the lowest Rg value of 1.91 to 1.98 nm (Fig. 8E–H).

Hydrogen bond is a robust non-covalent binding interaction, of which number in the compound-target complex indicates the strength of the binding. It was observed that CR45, CR46, CR52, and reynosin had hydrogen bond interactions with their corresponding targets during the entire simulation, which greatly contributed to the stability of the complexes (Fig. 8I–L).

The root mean square function (RMSF) is crucial for indicating the amino acid residues that undergo conformational changes within the protein. Peaks in the RMSF plot highlight the residues with pronounced oscillation during the simulation, suggesting greater flexibility in certain domains of the protein. It was shown that the residues at the location of 570, 643, 687, and 728 had greater fluctuation than other residues in the JAK2 when binding with CR45. After binding with CR52, greater fluctuation could be observed in the residues at site of 115, 181, 357, and 424 in SRC. The overall conformational changes within MAPK3 were insignificant, indicating the overall rigidity of the protein, while obvious changes were observed in the residues at the end of amino acid chain in the EGFR (Fig. 8M–P).

Binding free energy of compounds to their targets was further computed to depict the binding affinity. The binding free energy in the CR45-JAK2, CR46-MAPK3, CR52-SRC, and reynosin-EGFR complexes were–37.68 ± 5.86, –27.46 ± 3.51, –20.92 ± 5.23, and –26.8 ± 5.07 kcal/mol, respectively. The energy decomposition diagram provides details in the amino acid residues which have major contribution to the binding. The key residues in the binding between CR45 and JAK2 included Leu^551^ and Ile^559^. In the CR46-MAPK3 complex, Leu^107^ contributed greatly to the binding. Arg^160^ and Gln^362^ were main contributors in the CR52-SRC complex, while Gln^194^ made a great contribution to the binding between reynosin and EGFR (Fig. 8Q–T). These results showed that these key compounds in CR were able to form stable binding complex with their protein targets, which suggested their potential use in the treatment of NSCLC.

## 4. Discussion

The morbidity and mortality of NSCLC bring a heavy burden to the human health worldwide, highlighting the significance of understanding the pathogenesis of the disease and developing effective therapeutic approaches. CR, a common Chinese herb with evident anticancer bioactivity, has aroused our interest. Network pharmacology is a useful approach for exploring the underlying mechanism of drug bioactivity, based on the integration of network analysis, computational biology, and multidirectional pharmacology.^[19]^ In this study, network pharmacology was used to explore the substantial basis and mechanism underlying the therapeutic effect of CR on NSCLC.

We found that some terpenoids and diarylheptanoids were key chemical compounds in CR responsible for the treatment of NSCLC, highlighted by their high value of Degree in the compound-target network and virtually predicted bioactivity scores. According to the molecular docking, we found high binding affinity and various interactions in some complexes of compounds and critical targets, for example, (3*R*)-1-(3,4-dihydroxyphenyl)-7-phenyl-(6*E*)-6-hepten-3-ol and JAK2 (−8.1 kcal/mol) or (*E*)-1,7-bis(4-hydroxyphenyl)-6-hepten-3-one and SRC (−7.9 kcal/mol). Interestingly, these diarylheptanoids have a chemical structure similar to that of curcumin which had the highest Degree among the CR compounds in this study. In addition to CR, curcumin is also isolated from *Curcumae Longae Rhizoma* and *Curcumae Radix*. As one of the most studied natural products, curcumin is well-known for its pleiotropic roles in a series of diseases, including cancer, cardiovascular disease, diabetes, acquired immunodeficiency syndrome, gastric ulcer, psoriasis, arthritis, ulcerative colitis, tropical pancreatitis, and irritable bowel disease.^[20]^ Clinical trials have been extensively conducted to evaluate the potential of curcumin as human therapeutics.^[21]^ In terms of mechanism of anticancer bioactivity in lung, increasing in vivo or in vitro evidence suggests that treatment of lung cancer by curcumin involves various biological processes, such as suppressing cell proliferation, inducing apoptosis, inhibiting cell invasion and metastasis, triggering epigenetic changes, and modulating microRNA expression, via regulation of multiple targets.^[22]^ For instance, curcumin downregulates the phosphorylation of JAK2 and STAT3 and the expression of c-myc and cyclin D1 in a dose- and time-dependent manner, which inhibits the cell proliferation and colony formation in NCI-H460 lung cancer cells.^[23]^ Curcumin also decreases the phosphorylation of Akt in the PI3K/Akt/mTOR signaling pathway, thus inducing apoptosis in A549 and H1299 NSCLC cells.^[24]^ Importantly, some diarylheptanoids with a chemical structure similar to that of curcumin were identified as key compounds in CR treating NSCLC, which brings new insight into drug development in this series of analogs with promising therapeutic value in clinical applications. The application of curcumin is limited because of its poor solubility and bioavailability, and this issue may be addressed by structural modification with diarylheptanoids as lead compounds, thus expanding the horizon in the pharmaceutical industry.^[25]^ Exemplified by demethoxycurcumin, a curcuminoid with fewer methoxy groups than curcumin, exhibits increased stability and better aqueous solubility at physiological pH than curcumin.^[26]^ Intriguingly, this improved property of demethoxycurcumin may be associated with its superior efficacy to curcumin. It is shown that the hypomethylation effect of WIF-1 (a tumor suppressor gene) treated with demethoxycurcumin is stronger than that treated with curcumin in A549, SPC-A-1, and H460 cell lines.^[27]^ Apart from diarylheptanoids, sesquiterpenes are another large group of secondary metabolites in CR with various evident bioactivities.^[28,29]^ Our study suggested that reynosin, a sesquiterpene lactone, had high affinity to the critical targets of NSCLC, including EGFR, SRC, JAK2, and MAPK8, suggesting its therapeutic value in treating NSCLC. Although no study has focused on the treatment of lung cancer by reynosin, the cytotoxic effect of reynosin on human myeloid leukemia cells has been tested. Reynosin can inhibit the growth and cell viability of U937 and HL-60 cells by inducing apoptosis.^[30]^ In addition, both in vivo and in vitro evidence suggests that reynosin exerts hepatoprotective and anti-inflammatory effects, highlighting its potential use in treatment of cancer.^[31,32]^

In this study, some targets were found to play an essential role in the therapeutic effect of CR in NSCLC, among which EGFR had high values of topological parameters in PPI analysis and showed high affinity to the compounds in CR, suggesting its role as a key target in the anti-NSCLC bioactivity of CR. EGFR belongs to the ErbB family of receptor tyrosine kinases with crucial functions in epithelial cell physiology.^[33]^ Unstimulated EGFR is located at the plasma membrane in an auto-inhibited and dimerization-incompetent state. When binding to ligands, dimerization is triggered, allowing a series of structural rearrangements to be conveyed to the cytoplasmic domain and forming asymmetric dimers between 2 juxtaposed catalytic domains. These events cause allosteric activation of EGFR kinase and trans-autophosphorylation of tyrosine residues in the cytoplasmic receptor tail, thereby triggering a signaling cascade.^[34]^ In several human cancers, EGFR signaling is abnormally altered by gene amplification and/or protein overexpression, mutation, or in-frame deletion.^[35]^ Somatic EGFR activating mutations have been observed in approximately 15% to 20% of NSCLC patients.^[36]^ Upregulated levels of EGFR have been detected in the mitochondria in NSCLC cells. These organelles were redistributed to lamellipodia, and cell motility was increased by artificial mitochondria-targeted EGFR.^[37]^ Given the essential role of EGFR in lung cancer, EGFR-targeted therapies have been extensively developed, including monoclonal humanized antibodies against the extracellular domain of the receptor and selective small-molecule tyrosine kinase inhibitors with well-established efficacy (e.g., gefitinib, erlotinib, and afatinib).^[38]^ Unexpectedly, EGFR-targeted therapies have been found to respond in a limited manner and to evoke resistance frequently in patients mainly due to secondary mutations (e.g., T790M in NSCLC), alterations in other kinases, or feedback regulatory loops and mechanisms overcoming EGFR kinase inhibition.^[39]^ Moreover, analysis of PPI, transcription factor, and KEGG pathway enrichment suggested that the treatment of NSCLC with CR was strongly associated with the JAK2/STAT3 signaling pathway, a critical signal transduction pathway that plays an essential role in tumorigenesis and development.^[40]^ STAT3 is a core component of the JAK-STAT pathway and acts as a transcription factor downstream of various cytokines, hormones, interferons, and growth factors. When extracellular ligands bind to cognate receptors, canonical STAT3 signaling initiates and cause receptor dimerization and trans-phosphorylation of JAKs. The activation of JAKs leads to phosphorylation of cytoplasmic receptor-tails, forming docking sites for STAT3. Subsequently, STAT3 is activated by phosphorylation of a single tyrosine residue on Tyr^705^ induced by JAKs. Once activated, STAT3 dissociates from the receptor-kinase complex and forms homo- or hetero-dimers. STAT3 dimers eventually translocate into the nucleus and trigger the transcription of genes associated with a series of cancer hallmarks, including proliferation, apoptosis, angiogenesis, and immune evasion.^[41]^ Non-receptor tyrosine kinases, such as SRC, can also activate the JAK/STAT3 signaling pathway.^[42]^ In samples from NSCLC patients and cell lines, persistent STAT3 activation can be observed, and high expression of intratumoral phosphorylated STAT3 correlates with an advanced disease stage and EGFR mutation. Accumulating evidence suggests that the hyperactivation of STAT3 within tumors and immune cells shapes the tumor microenvironment and STAT3 causes tumor-promoting inflammation while impairing antitumor immunity.^[43,44]^ Although STAT3-targeted therapeutics have been developed, challenges continue to emerge. Usually, the favorable performance of strategies to inhibit upstream components of STAT3 signaling in preclinical studies cannot be recapitulated in patients with NSCLC, especially when the disease stage is advanced. In addition, STAT3-inhibiting therapy could be limited due to accompanying adverse drug reactions, including infections, rectal hemorrhage, abnormal liver function, thrombocyto- and neutropenia, anemia, and neurological disorders.^[41]^ Therefore, there is an urgent need to exploit new therapeutic targeting core components in development of NSCLC with potent efficacy and safety in the clinic. Although our study is a in silico computational study, it provides new insights into drug development by exploring resources from natural products using a network pharmacology-based strategy. The data will be more convincing if a more comprehensive approach can be used, for example, the integration of bioinformatics, multiomics, and in vitro and in vivo study. And the combined therapy of NSCLC by CR with chemical agents is also promising, from which the patients with NSCLC in clinic will be greatly benefited.

## 5. Conclusion

In summary, we explored the key compounds and underlying mechanism of CR in the treatment of NSCLC. We found that reynosin and diarylheptanoids were the key chemical compounds responsible for the anti-NSCLC bioactivity of CR. The therapeutic potential of CR was closely associated with the modulation of critical targets, such as EGFR, JAK2, SRC, and MAPK3. This study provides a useful strategy for exploring new therapeutic approaches for NSCLC using Chinese herbal medicines.

## Author contributions

**Conceptualization:** Zhirui Yang, Mingquan Wu, Lin Li.

**Data curation:** Zhirui Yang, Xin Zhou.

**Formal analysis:** Zhirui Yang, Jin Luo.

**Funding acquisition:** Mingquan Wu.

**Investigation:** Zhirui Yang, Yi Liu.

**Methodology:** Zhirui Yang.

**Project administration:** Zhirui Yang.

**Resources:** Zhirui Yang.

**Software:** Zhirui Yang, Xin Zhou, Jin Luo.

**Supervision:** Mingquan Wu, Lin Li.

**Validation:** Yi Liu.

**Visualization:** Zhirui Yang.

**Writing – original draft:** Zhirui Yang.

**Writing – review & editing:** Zhirui Yang, Mingquan Wu, Lin Li.

## Supplementary Material


