# Supplementary figures and images for: Network pharmacology study on the mechanism of Curcumae Rhizoma in the treatment of non-small cell lung cancer
Source: Medicine (Baltimore). 2025 May 9;104(19):e42366. doi: 10.1097/MD.0000000000042366 (PMC12074036; doi:10.1097/MD.0000000000042366)

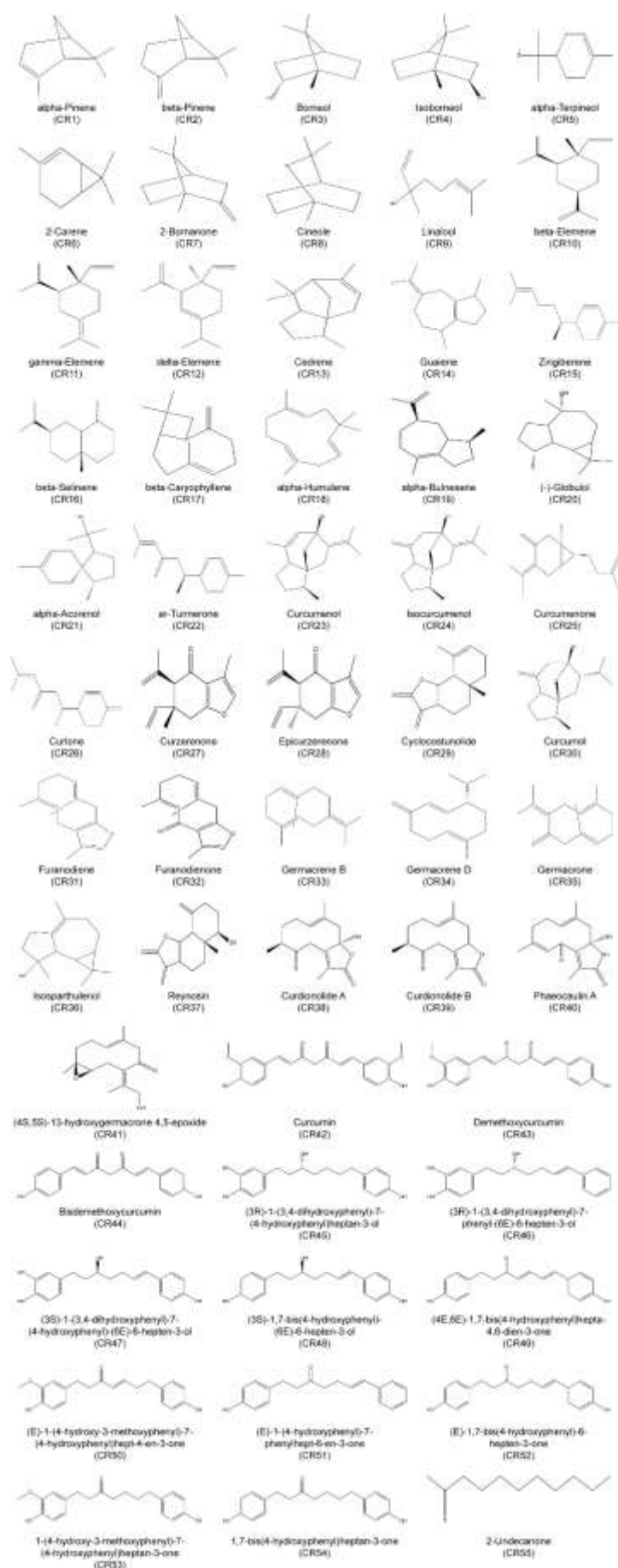

**S\_Figure 1.** Representative compounds in *Curcuma Rhizoma*. CR, *Curcuma Rhizoma*.

Supplement: Supplementary file 1 [file medi-104-e42366-s001.pdf]
