# Supplementary material for: Network pharmacology study on the mechanism of Curcumae Rhizoma in the treatment of non-small cell lung cancer
Source: Medicine (Baltimore). 2025 May 9;104(19):e42366. doi: 10.1097/MD.0000000000042366 (PMC12074036; doi:10.1097/MD.0000000000042366)
Supplement: Supplementary file 2 [file medi-104-e42366-s002.pdf]

S_Table 2. Topological parameters of *Curcumae Rhizoma* compounds in the compound-target network

Compounds	Betweenness Centrality	Closeness Centrality	Degree	Average Shortest Path Length
CR42	0.7205265	0.65677966	89	1.52258065
CR54	0.06533983	0.40789474	16	2.4516129
CR44	0.01670302	0.3490991	14	2.86451613
CR41	0.03868466	0.39540816	12	2.52903226
CR53	0.02277683	0.35067873	12	2.8516129
CR51	0.02748826	0.39540816	11	2.52903226
CR40	0.02415225	0.37621359	10	2.65806452
CR50	0.00919179	0.32157676	9	3.10967742
CR45	0.02102863	0.38557214	8	2.59354839
CR10	0.00655641	0.3587963	6	2.78709677
CR43	0.00481283	0.3587963	6	2.78709677
CR37	0.00427035	0.36046512	5	2.77419355
CR39	0.01589421	0.36384977	5	2.7483871
CR49	0.00163034	0.31	5	3.22580645
CR30	0.01406892	0.33119658	4	3.01935484
CR38	6.70E-04	0.32157676	4	3.10967742
CR18	0.00208003	0.35388128	3	2.82580645
CR22	0.00520327	0.37439614	3	2.67096774
CR46	9.92E-04	0.32157676	3	3.10967742
CR52	0.00301187	0.3587963	3	2.78709677
CR5	6.40E-04	0.35227273	2	2.83870968
CR7	1	1	2	1
CR8	0.00119243	0.35067873	2	2.8516129
CR12	0.00119243	0.35067873	2	2.8516129
CR17	0.00119243	0.35067873	2	2.8516129

CR23	1.00E-04	0.31376518	2	3.18709677
CR24	0.00119243	0.35067873	2	2.8516129
CR27	7.60E-04	0.35388128	2	2.82580645
CR28	7.60E-04	0.35388128	2	2.82580645
CR31	9.08E-04	0.35388128	2	2.82580645
CR32	9.08E-04	0.35388128	2	2.82580645
CR55	6.52E-04	0.35550459	2	2.81290323
CR1	0	0.31762295	1	3.1483871
CR2	0	0.3490991	1	2.86451613
CR3	0	0.3490991	1	2.86451613
CR4	0	0.3490991	1	2.86451613
CR6	0	0.31762295	1	3.1483871
CR9	0	0.3490991	1	2.86451613
CR11	0	0.3490991	1	2.86451613
CR13	0	0.3490991	1	2.86451613
CR14	0	0.3490991	1	2.86451613
CR15	0	0.3490991	1	2.86451613
CR16	0	0.3490991	1	2.86451613
CR19	0	0.31762295	1	3.1483871
CR20	0	0.31762295	1	3.1483871
CR21	0	0.31762295	1	3.1483871
CR25	0	0.3490991	1	2.86451613
CR26	0	0.3490991	1	2.86451613
CR29	0	0.3490991	1	2.86451613
CR33	0	0.3490991	1	2.86451613
CR34	0	0.3490991	1	2.86451613
CR35	0	0.3490991	1	2.86451613
CR36	0	0.31762295	1	3.1483871
CR47	0	0.23065476	1	4.33548387

CR48	0	0.31762295	1	3.1483871

Abbreviation: CR, *Curcumae Rhizoma*.
