# Supplementary material for: Network pharmacology study on the mechanism of Curcumae Rhizoma in the treatment of non-small cell lung cancer
Source: Medicine (Baltimore). 2025 May 9;104(19):e42366. doi: 10.1097/MD.0000000000042366 (PMC12074036; doi:10.1097/MD.0000000000042366)
Supplement: Supplementary file 3 [file medi-104-e42366-s003.pdf]

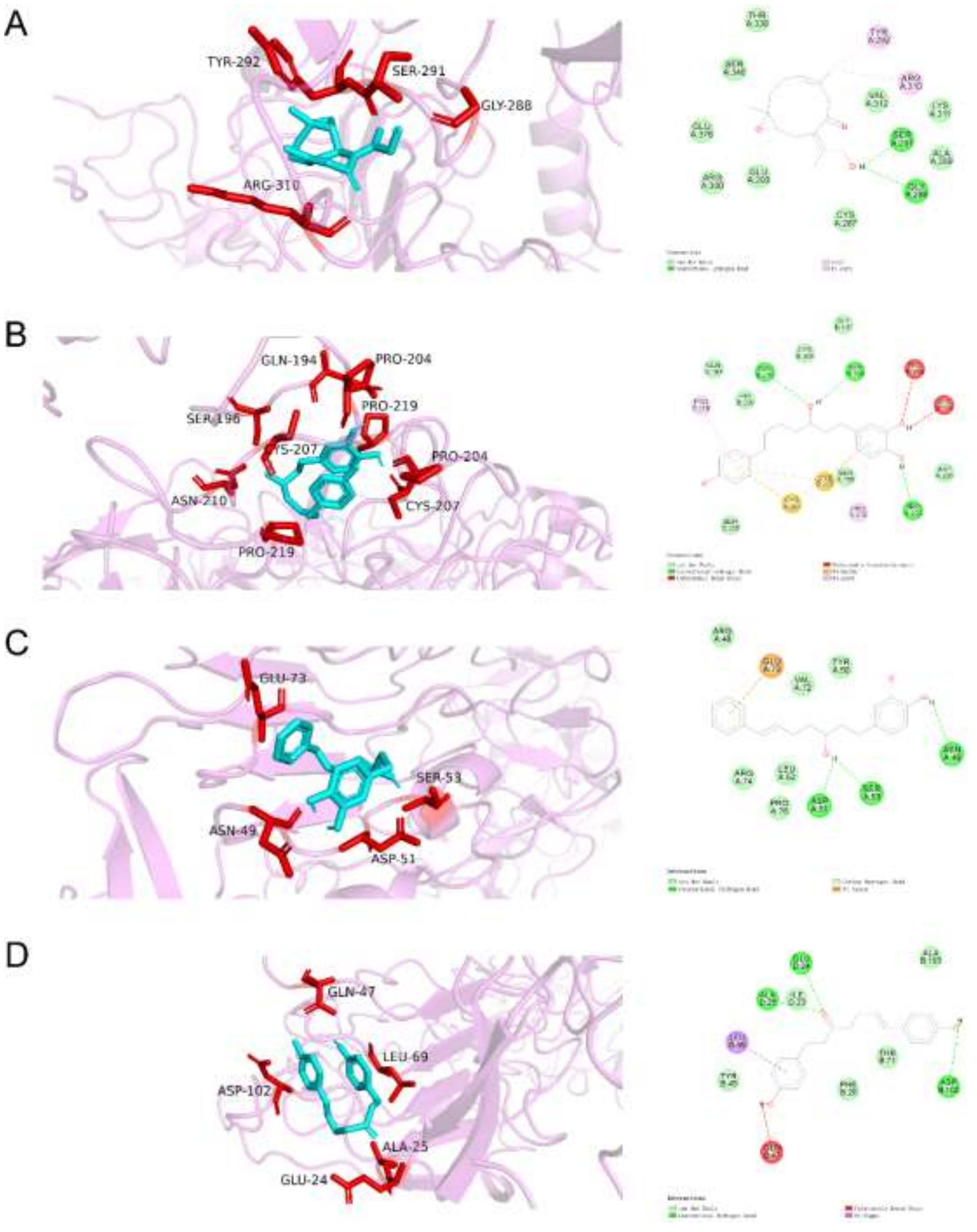

S_Figure 2. Molecular docking of EGFR and representative compounds in *Curcumae Rhizoma*. (A) EGFR and (4*S*,5*S*)-13-hydroxygermacrone 4,5-epoxide. (B) EGFR and (3*R*)-1-(3,4-dihydroxyphenyl)-7-(4-hydroxyphenyl)heptan-3-ol. (C) EGFR and (3*R*)-1-(3,4-dihydroxyphenyl)-7-phenyl-(6*E*)-6-hepten-3-ol (D) EGFR and (*E*)-1,7-bis(4-hydroxyphenyl)-6-hepten-3-one.

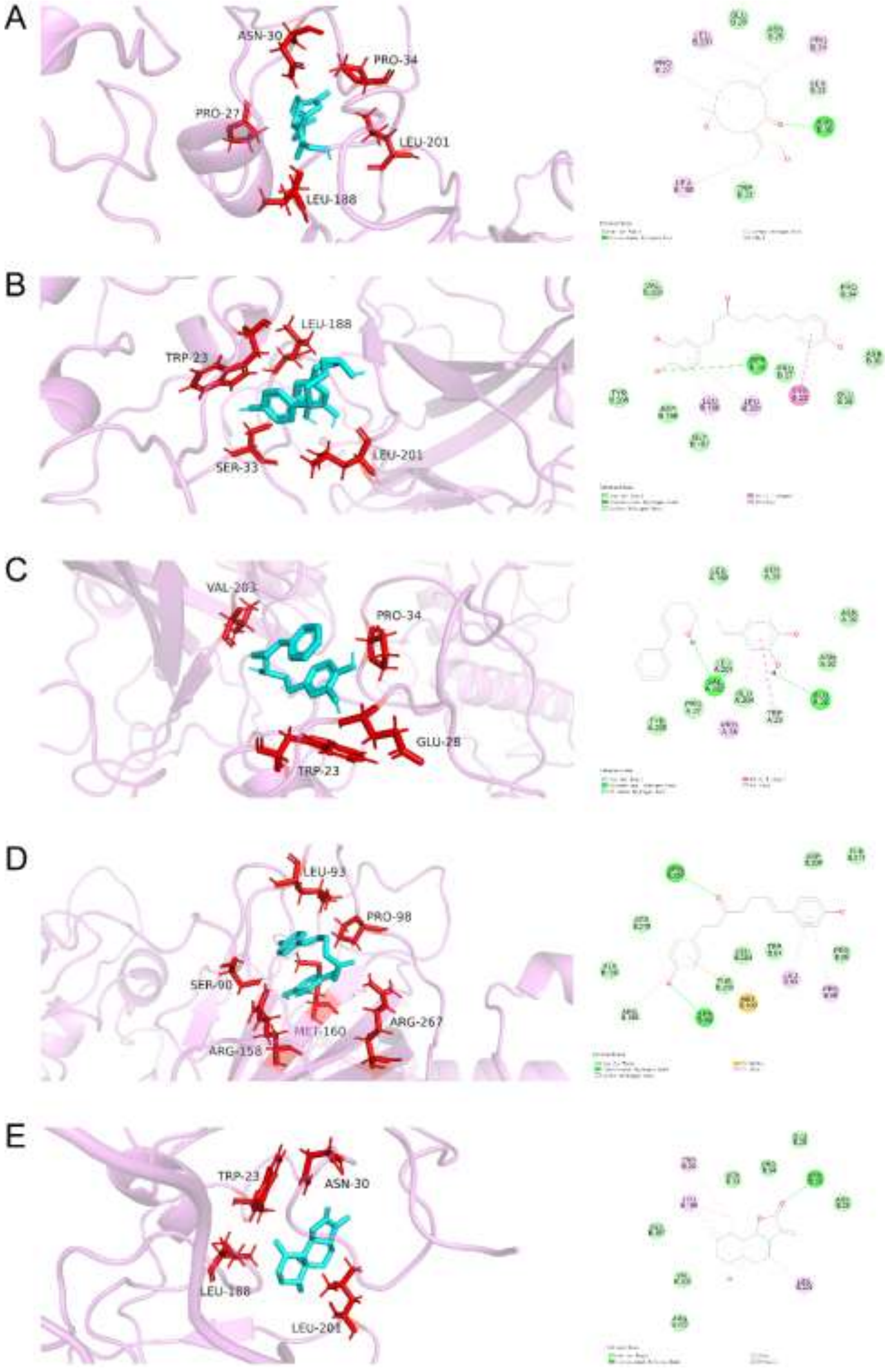

S_Figure 3. Molecular docking of TP53 and representative compounds in *Curcumae Rhizoma*. (A) TP53 and (4*S*,5*S*)-13-hydroxygermacrone 4,5-epoxide. (B) TP53 and(3*R*)-1-(3,4-dihydroxyphenyl)-7-(4-hydroxyphenyl)heptan-3-ol. (C) TP53 and (3*R*)-1-(3,4-dihydroxyphenyl)-7-phenyl-(6*E*)-6-hepten-3-ol. (D) TP53 and (*E*)-1,7-bis(4-hydroxyphenyl)-6-hepten-3-one. (E) TP53 and reynosin.

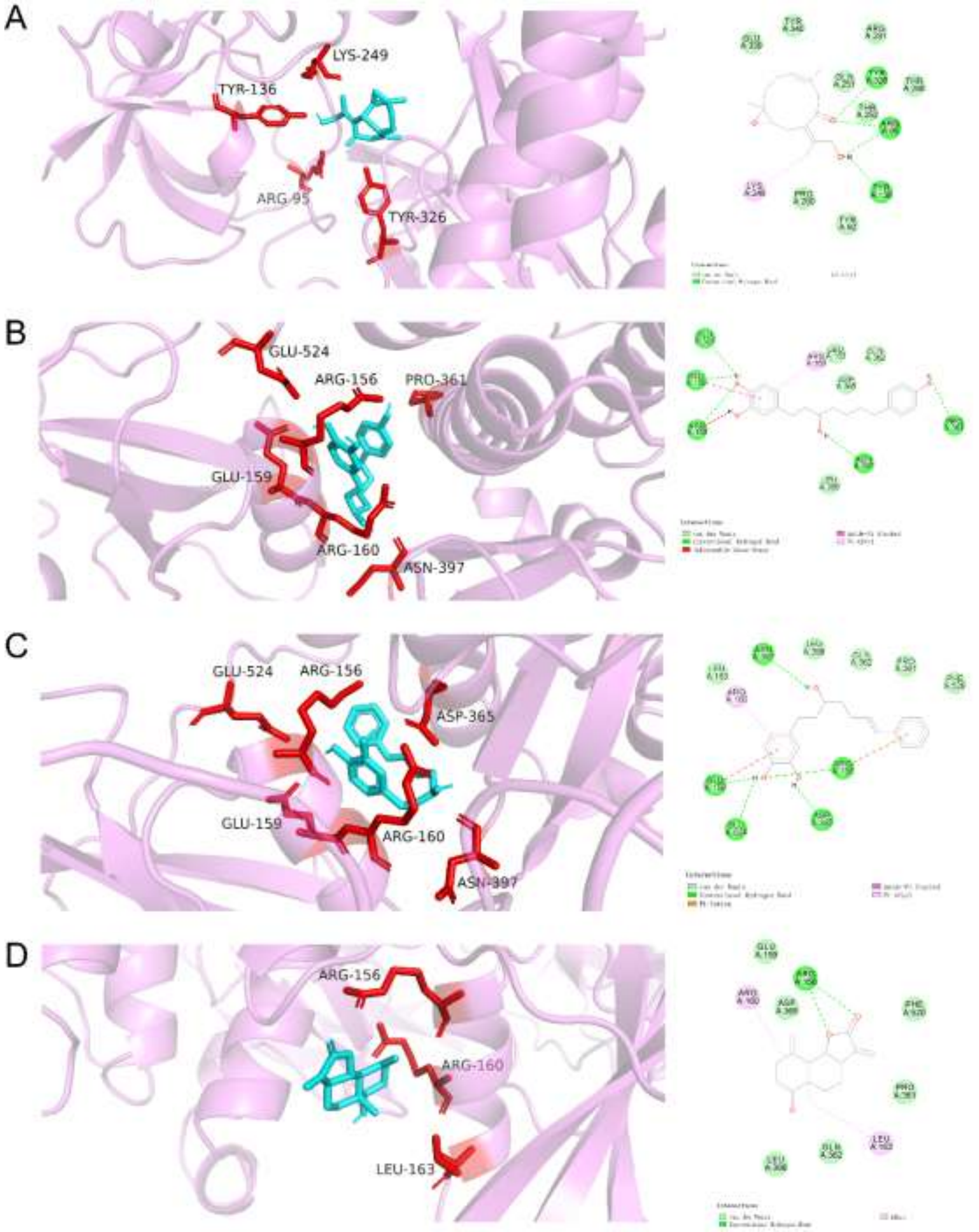

S_Figure 4. Molecular docking of SRC and representative compounds in *Curcumae Rhizoma*. (A) SRC and (4*S*,5*S*)-13-hydroxygermacrone 4,5-epoxide. (B) SRC and (3*R*)-1-(3,4-dihydroxyphenyl)-7-(4-hydroxyphenyl)heptan-3-ol. (C) SRC and (3*R*)-1-(3,4-dihydroxyphenyl)-7-phenyl-(6*E*)-6-hepten-3-ol. (D) SRC and reynosin.

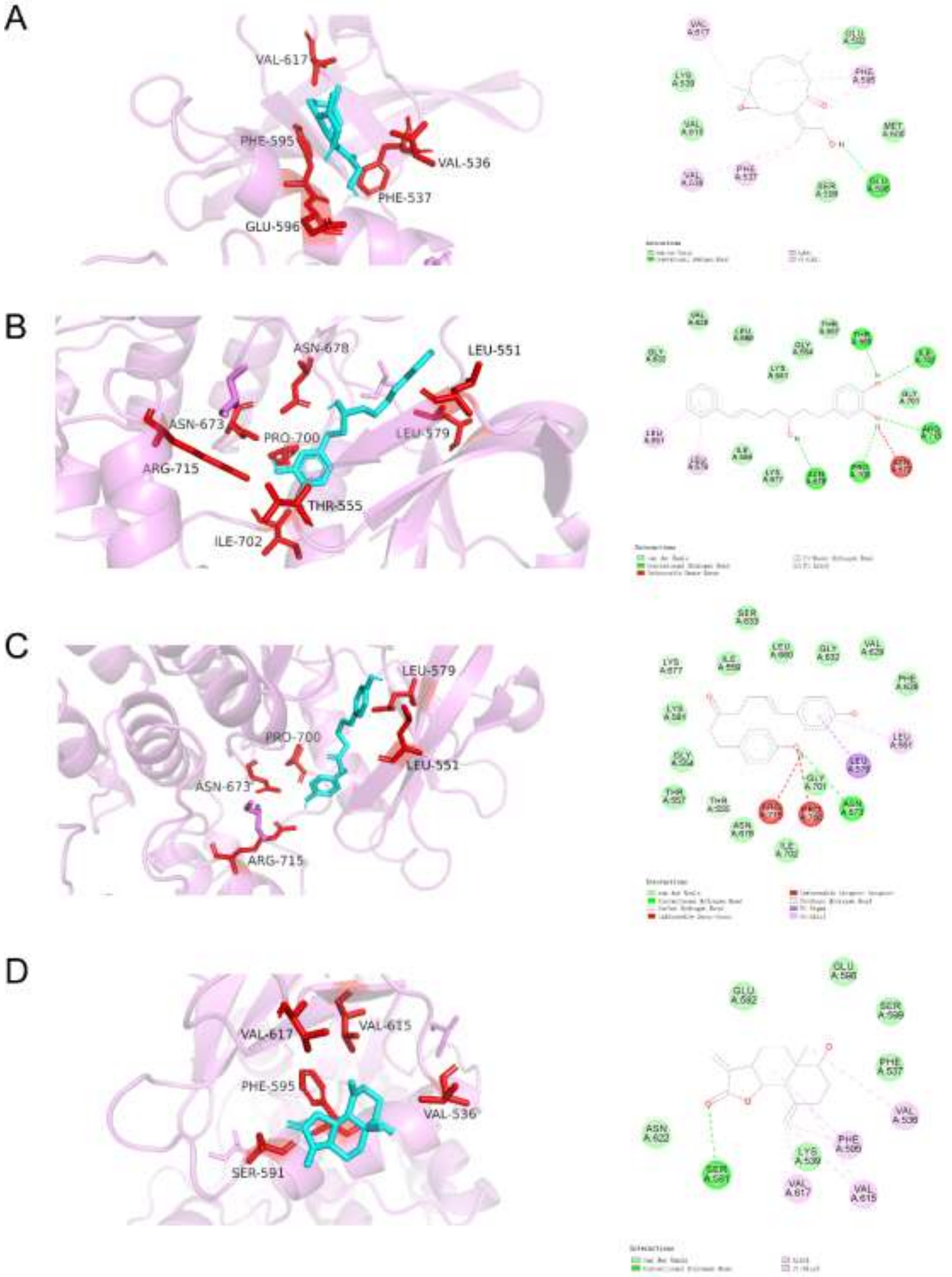

S_Figure 5. Molecular docking of JAK2 and representative compounds in *Curcumae Rhizoma*. (A) JAK2 and (4*S*,5*S*)-13-hydroxygermacrone 4,5-epoxide. (B) JAK2 and (3*R*)-1-(3,4-dihydroxyphenyl)-7-phenyl-(6*E*)-6-hepten-3-ol. (C) JAK2 and (*E*)-1,7-bis(4-hydroxyphenyl)-6-hepten-3-one. (D) JAK2 and reynosin.

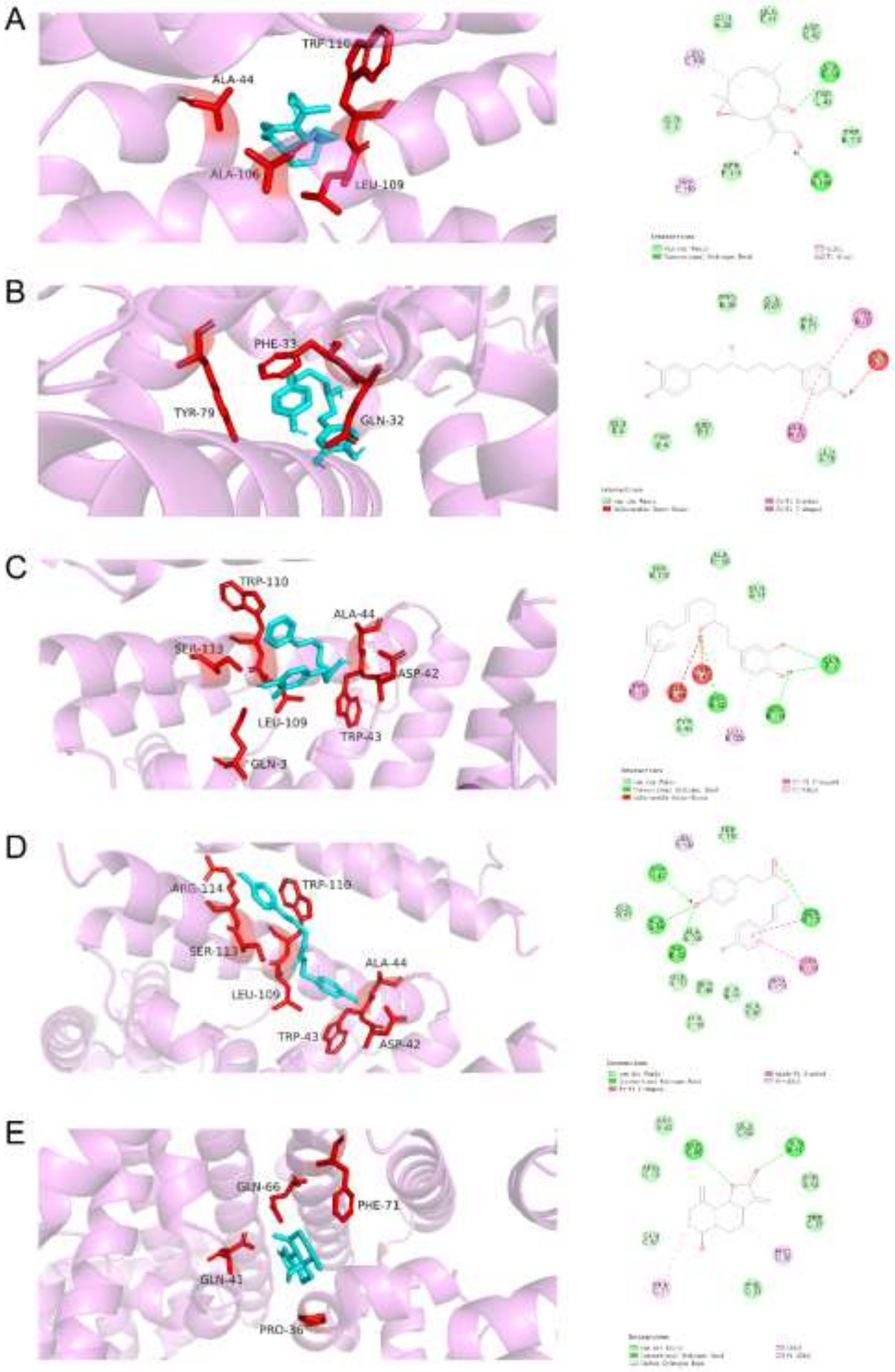

S_Figure 6. Molecular docking of STAT3 and representative compounds in *Curcumae Rhizoma*. (A) STAT3 and (4*S*,5*S*)-13-hydroxygermacrone 4,5-epoxide. (B) STAT3 and (3*R*)-1-(3,4-dihydroxyphenyl)-7-(4-hydroxyphenyl)heptan-3-ol. (C) STAT3 and (3*R*)-1-(3,4-dihydroxyphenyl)-7-phenyl-(6*E*)-6-hepten-3-ol. (D) STAT3 and (*E*)-1,7-bis(4-hydroxyphenyl)-6-hepten-3-one. (E) STAT3 and reynosin.

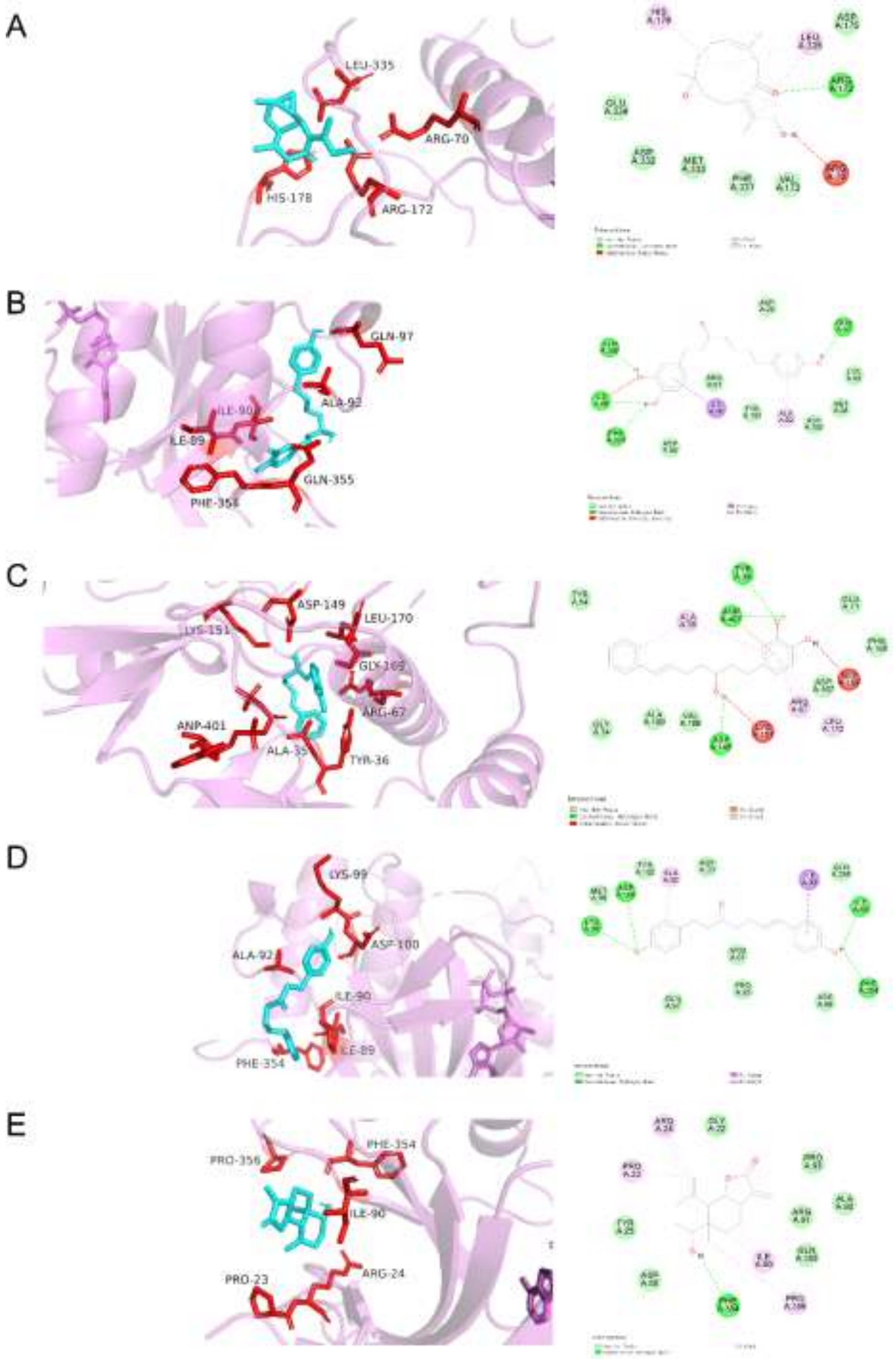

S_Figure 7. Molecular docking of MAPK1 and representative compounds in *Curcumae Rhizoma*. (A) MAPK1 and (4*S*,5*S*)-13-hydroxygermacrone 4,5-epoxide. (B) MAPK1 and (3*R*)-1-(3,4-dihydroxyphenyl)-7-(4-hydroxyphenyl)heptan-3-ol. (C) MAPK1 and (3*R*)-1-(3,4-dihydroxyphenyl)-7-phenyl-(6*E*)-6-hepten-3-ol. (D) MAPK1 and (*E*)-1,7-bis(4-hydroxyphenyl)-6-hepten-3-one. (E) MAPK1 and reynosin.

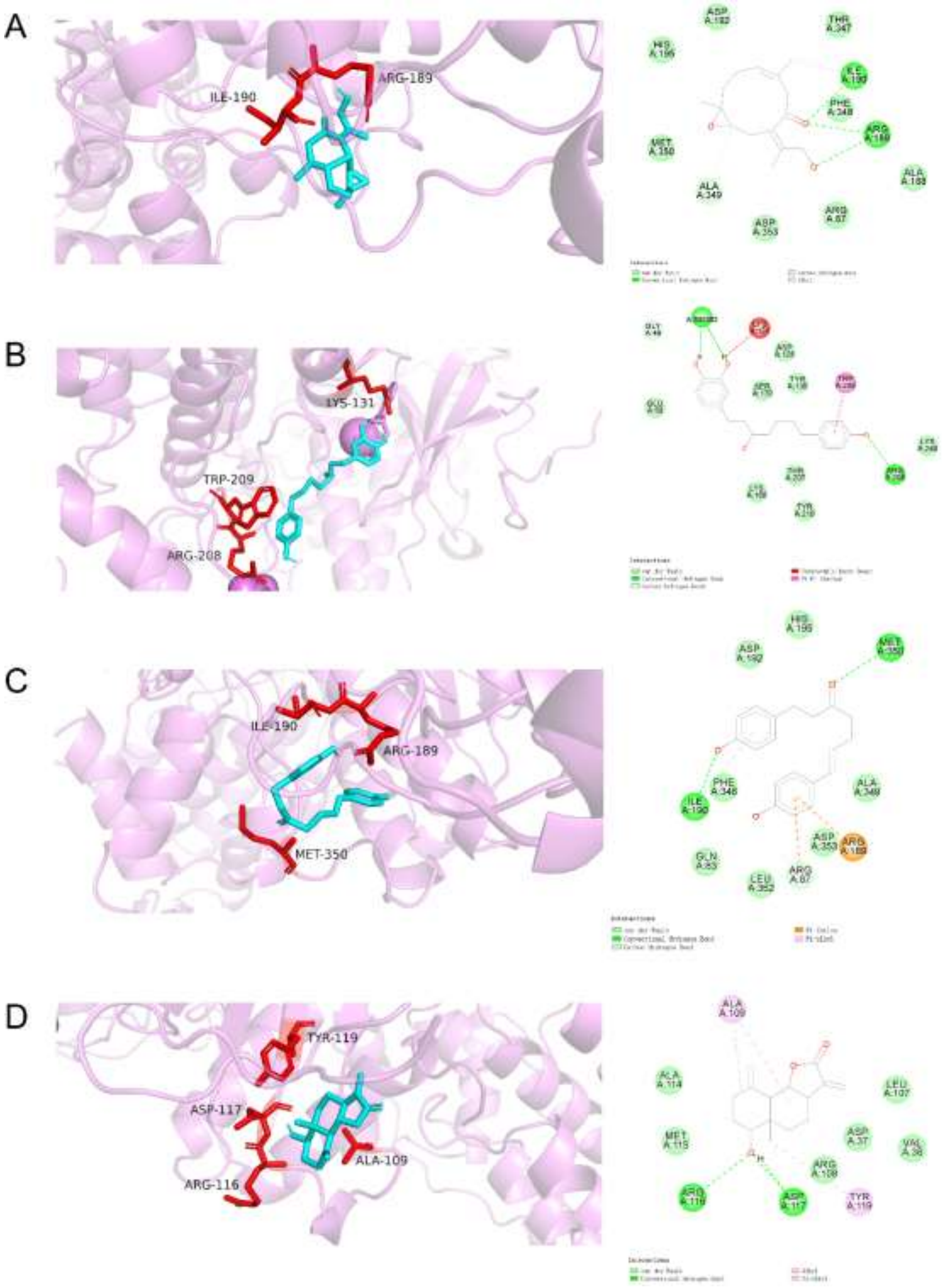

S_Figure 8. Molecular docking of MAPK3 and representative compounds in *Curcumae Rhizoma*. (A) MAPK3 and (4*S*,5*S*)-13-hydroxygermacrone 4,5-epoxide. (B) MAPK3 and (3*R*)-1-(3,4-dihydroxyphenyl)-7-(4-hydroxyphenyl)heptan-3-ol. (C) MAPK3 and (*E*)-1,7-bis(4-hydroxyphenyl)-6-hepten-3-one. (D) MAPK3 and reynosin.

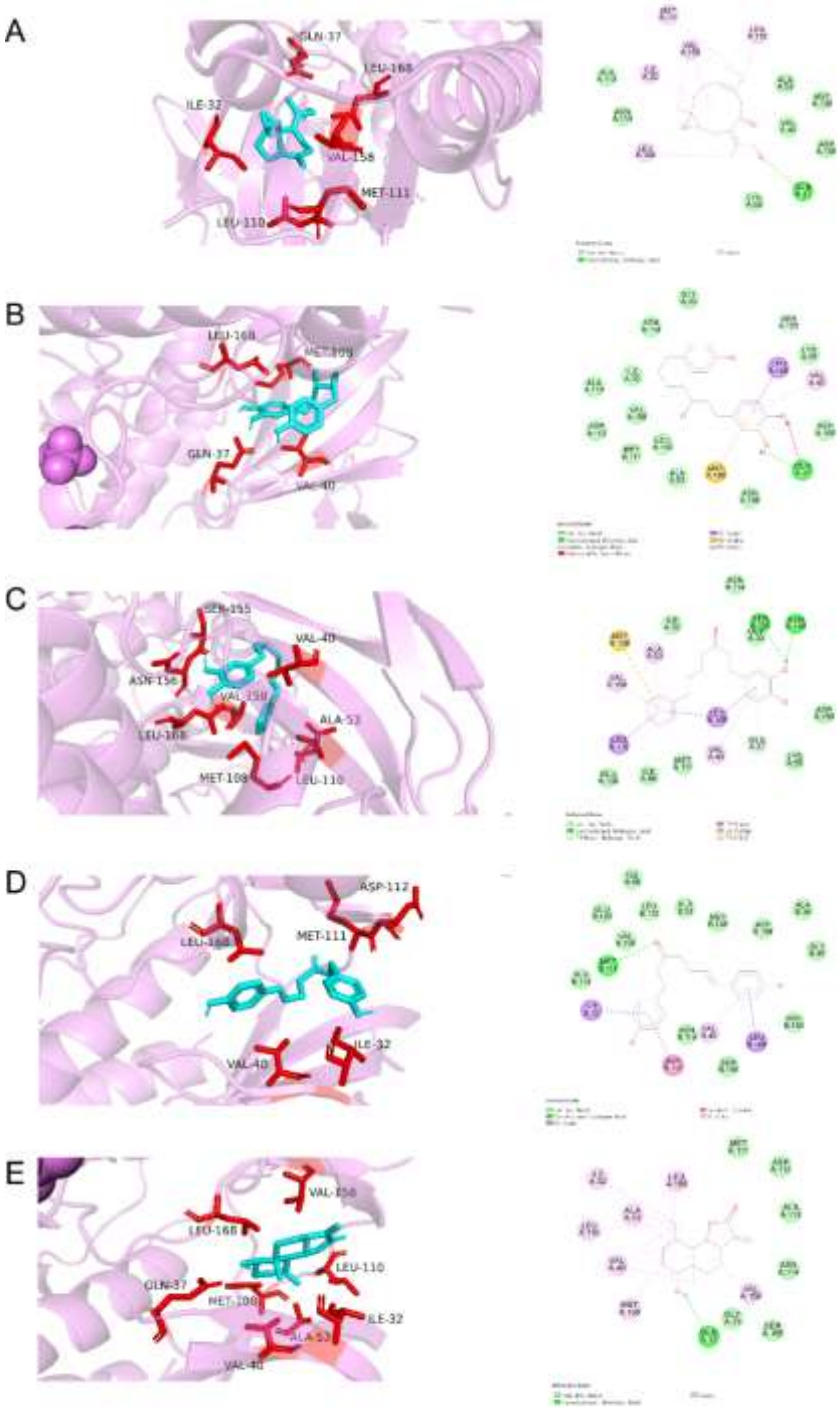

S_Figure 9. Molecular docking of MAPK8 and representative compounds in *Curcumae Rhizoma*. (A) MAPK8 and (4*S*,5*S*)-13-hydroxygermacrone 4,5-epoxide. (B) MAPK8 and (3*R*)-1-(3,4-dihydroxyphenyl)-7-(4-hydroxyphenyl)heptan-3-ol. (C) MAPK8 and (3*R*)-1-(3,4-dihydroxyphenyl)-7-phenyl-(6*E*)-6-hepten-3-ol. (D) MAPK8 and (*E*)-1,7-bis(4-hydroxyphenyl)-6-hepten-3-one. (E) MAPK8 and reynosin.

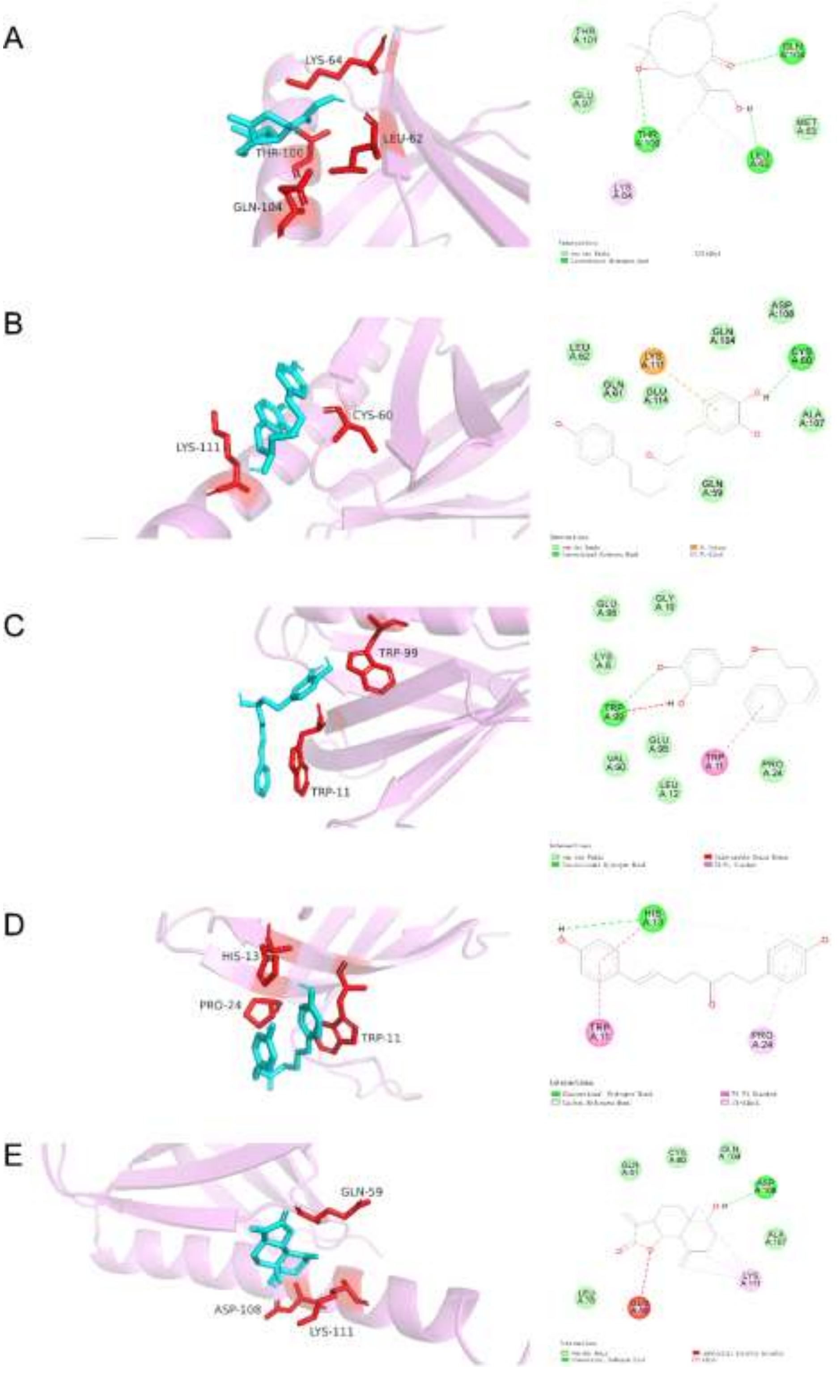

S_Figure 10. Molecular docking of AKT1 and representative compounds in *Curcumae Rhizoma*. (A) AKT1 and (4*S*,5*S*)-13-hydroxygermacrone 4,5-epoxide. (B) AKT1 and (3*R*)-1-(3,4-dihydroxyphenyl)-7-(4-hydroxyphenyl)heptan-3-ol. (C) AKT1 and (3*R*)-1-(3,4-dihydroxyphenyl)-7-phenyl-(6*E*)-6-hepten-3-ol. (D) AKT1 and (*E*)-1,7-bis(4-hydroxyphenyl)-6-hepten-3-one. (E) AKT1 and reynosin.

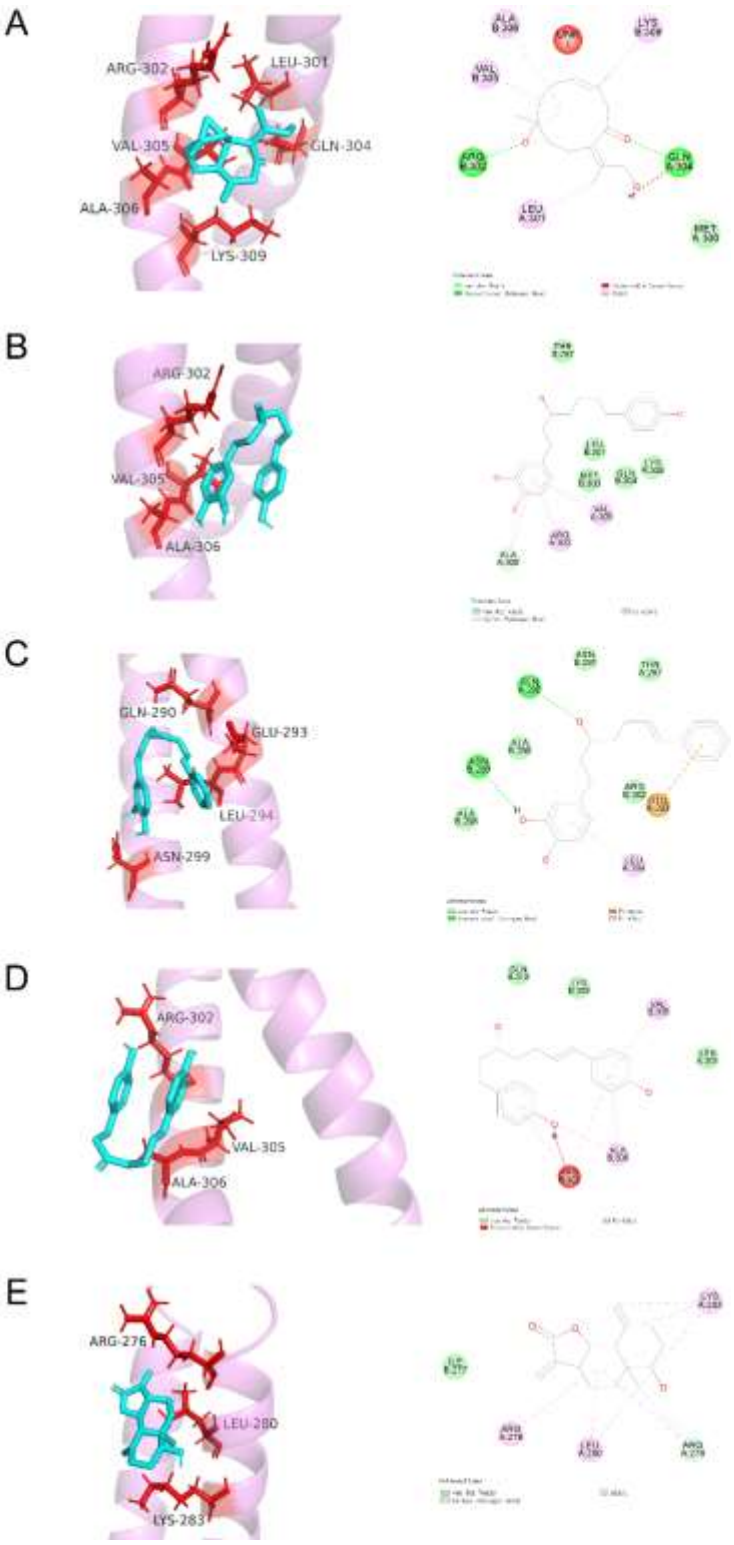

S_Figure 11. Molecular docking of JUN and representative compounds in *Curcumae Rhizoma*. (A) JUN and (4*S*,5*S*)-13-hydroxygermacrone 4,5-epoxide. (B) JUN and (3*R*)-1-(3,4-dihydroxyphenyl)-7-(4-hydroxyphenyl)heptan-3-ol. (C) JUN and (3*R*)-1-(3,4-dihydroxyphenyl)-7-phenyl-(6E)-6-hepten-3-ol. (D) JUN and (*E*)-1,7-bis(4-hydroxyphenyl)-6-hepten-3-one. (E) JUN and reynosin.
